# Electron Donor–Acceptor Interface of TPPS/PDI Boosting Charge Transfer for Efficient Photocatalytic Hydrogen Evolution

**DOI:** 10.1002/advs.202201134

**Published:** 2022-04-11

**Authors:** Jun Yang, Jianfang Jing, Wenlu Li, Yongfa Zhu

**Affiliations:** ^1^ Department of Chemistry Tsinghua University Beijing 100084 P. R. China

**Keywords:** charge separation, electron transfer, interfacial electric field, photocatalytic hydrogen production, TPPS/PDI

## Abstract

Charge separation efficiency of photocatalysts is still the key scientific issue for solar‐to‐chemical energy conversion. In this work, an electron donor–acceptor (D‐A) interface with high charge separation between TPPS (tetra(4‐sulfonatophenyl)porphyrin) and PDI (perylene diimide) is successfully constructed for boosting photocatalytic H_2_ evolution. The TPPS/PDI with D‐A interface shows excellent photocatalytic H_2_ evolution rate of 546.54 µmol h^–1^ (30.36 mmol h^–1^ g^–1^), which is 9.95 and 9.41 times higher than that of pure TPPS and PDI, respectively. The TPPS/PDI has a markedly stronger internal electric field, which is respectively 3.76 and 3.01 times higher than that of pure PDI and TPPS. The D‐A interface with giant internal electric field efficiently facilitates charge separation and urges TPPS/PDI to have a longer excited state lifetime than single component. The work provides entirely new ideas for designing materials with D‐A interface to realize high photocatalytic activity.

## Graphical abstract

A novel electron donor–acceptor (D‐A) interface between TPPS and PDI is successfully constructed. The D‐A interface of TPPS/PDI dramatically promotes the charge separation and extends the lifetime of the excited state. As a result, TPPS/PDI exhibits a high‐efficiency H_2_ evolution activity with outstanding rate of 546.54 µmol h^‐1^, surpassing many reported organic photocatalysts.

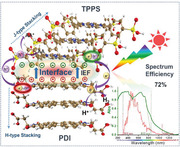

## 1 Introduction

Semiconductor‐based photocatalytic hydrogen production technology plays an important role in solving environmental and energy crises and has become a research hotspot.^[^ ^1^ ^]^ Despite a great deal of work has been done in the development of highly efficient photocatalytic hydrogen evolution materials, it still suffers from the limited solar spectral response and the rapid recombination of photogenerated electrons and holes, which severely limits the industrial application of hydrogen production.^[^ ^2^ ^]^ Hence, developing materials with broad spectral response and improving charge separation efficiency are the key scientific problems to enhance the performance of photocatalysts.^[^ ^3^ ^]^ It should be pointed out that, the internal electric field (IEF) is a powerful driving force for the separation of photogenerated charge, which is closely related to photocatalytic performance.^[^ ^4^ ^]^

Due to the structural diversity and adjustability, various conjugated organic materials show great potential in photocatalysis, which are promising as an alternative to inorganic materials for H_2_ evolution.^[^ ^5^ ^]^ Especially, porphyrin‐based and perylene‐based materials are drawing researchers’ great interest due to the excellent wide‐spectrum absorption and outstanding photoelectric conversion.^[^ ^6^ ^]^ Recently, several perylene‐based and porphyrin‐based materials have been developed by our group for photocatalytic hydrogen and oxygen production.^[^ ^7^ ^]^ In our studies, the intrinsic internal electric field of materials is often improved by increasing the molecular dipole, thus improving the photocatalytic performance. However, improving the intrinsic internal electric field of single‐component often involves very complex preparation processes and such an intrinsic IEF induced by large molecular dipole is still limited in improving charge separation efficiency. As a consequence, exploring new strategies to obtain giant IEF is necessary to achieve a breakthrough in photocatalytic performance.

Inspired by the excellent electron‐donor properties of porphyrin and the electron‐acceptor properties of perylene diimide, the idea of constructing an interface with electron donor–acceptor (D‐A) feature at the interface of TPPS and PDI came to mind.^[^ ^8^ ^]^ Furthermore, the study of interfacial internal electric field between TPPS and PDI has not been reported yet. To our knowledge, most porphyrins are commonly used as photosensitizer or building blocks of covalent organic frameworks and metal‐organic frameworks,^[^ ^9^ ^]^ rather than as photocatalysts for building interfacial electric field at the interface. Besides, the short‐range J‐type stacking of TPPS can well simulate the chlorophyll in nature.^[^ ^10^ ^]^ And that the long‐range H‐type stacking of PDI can provide long‐range electrons delocalization, which is benefit to building the charge transfer channels.^[^ ^7b^ ^]^ Integration of the two types of *π*–*π* stacking is hopeful for improving photocatalytic efficiency.

Based on the above analysis, the novel coassembly TPPS/PDI organic semiconductor with D‐A‐type interface is successfully designed through *π*–*π* stacking interaction for photocatalytic H_2_ evolution. TPPS/PDI has excellent spectral response, covering the entire solar spectrum (theoretical spectral efficiency: 72%). It is proved that the interface with D‐A feature between TPPS and PDI greatly benefits spatial charge separation. Meanwhile, the giant interfacial electric field at the interface dramatically extends the lifetime of charge separation state, which effectively promotes photogenerated electrons to participate in the reduction reaction. As a result, TPPS/PDI exhibits a much increased H_2_ evolution performance.

## 2 Results and Discussion

### 2.1 Construction of D‐A Interface between TPPS and PDI

**Figure 1:**
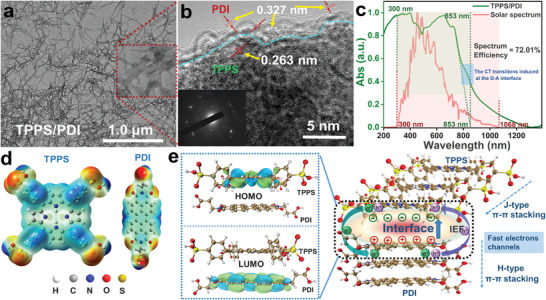
a) TEM image of TPPS/PDI. b) HRTEM image of TPPS/PDI. c) the UV–vis diffuse reflection spectroscopy of TPPS/PDI power, and solar spectrum observed by optical fiber spectrometer. d) The molecular formula and electrostatic potential distribution of TPPS and PDI. e) Left: the frontier molecular orbital distribution of TPPS and PDI at the interface; Right: schematic diagram of interfacial interaction of coassembly supramolecular TPPS/PDI.

First, the TPPS/PDI sample was successfully constructed via coassembly, which was described in detail in the experimental part. The TPPS/PDI (**Figure** **1**a, Figures S2 and S3, Supporting Information) mainly exhibited nanowires structure via *π*–*π* stacking by dissolution‐precipitation process. High‐resolution transmission electron microscopy (HRTEM) of Figure 1b showed that TPPS/PDI has high crystallinity, in which *d*‐spacing of 0.327 and 0.263 nm assigned to PDI and TPPS, respectively (Figure S2, Supporting Information). High crystallinity is conducive to the construction of strong interfacial electric field between TPPS and PDI, so that it has good charge separation capabilities.^[^ ^7^, ^11^ ^]^ As shown in Figure S4 (Supporting Information), the results of contact angles indicate TPPS, PDI, and TPPS/PDI have an excellent hydrophilicity due to the existence of carboxylic and sulfonic groups, which is favorable for catalytic reaction.

The distinguishable spectroscopic features can reflect the aggregation type of samples. The ultraviolet–visible (UV–vis) spectrum in Figure S5 (Supporting Information) shows that TPPS has typical Soret bands and Q bands. The redshifted Soret band and the reduced Q bands of TPPS indicate the formation of J‐type accumulation compared with the TPPS monomer.^[^ ^12^ ^]^ As shown in Figure S6 (Supporting Information), three distinct peaks of PDI monomer appear, corresponding electronic transitions of PDI molecules. However, as for PDI self‐assembly nanofibers, the blueshift and the disappearance of the fine curve structure of absorption peaks indicate the formation of H‐type accumulation.^[^ ^13^ ^]^ As shown in Figure S7 (Supporting Information), the UV–vis absorption spectra of TPPS/PDI shows that TPPS and PDI present J‐type and H‐type accumulation respectively. As for the coassembly TPPS/PDI, the short‐range J‐type stacking of TPPS can well simulate the chlorophyll in nature. And the long range H‐type stacking of PDI helps to provide larger electron delocalization and facilitate electron migration.^[^ ^7^, ^10^ ^]^ The typical XRD patterns of *d*‐spacing of *π*–*π* accumulation was shown in Figure S8 (Supporting Information).^[^ ^14^ ^]^ The electron spin resonance (ESR) technique furthermore confirmed the strong *π*–*π* interaction between PDI and TPPS (Figure S9, Supporting Information). Compared with physical mixture of TPPS and PDI, the generation of singlet oxygen of the coassembly TPPS/PDI exhibited an obvious decrease, which attributed to the quenching of the excitation energy by strong *π*–*π* stacking between TPPS and PDI.^[^ ^15^ ^]^

Besides, TPPS/PDI shows excellent the whole solar spectral response, whose theoretical spectral efficiency reaches up to 72% (Figure 1c). The outstanding light absorption of TPPS/PDI would highly contribute to improving the conversion of solar energy to chemical energy. Besides, in the solid‐state absorption spectra of both architectures, new absorptions emerge around 840 nm, which are assigned to charge transfer (CT) transitions (Figure 1c).^[^ ^16^ ^]^ The molecular formula and electrostatic potential distribution of TPPS and PDI are shown in Figure 1d. According to the results of theoretical calculation, the lowest unoccupied molecular orbital (LUMO) and the highest occupied molecular orbital (HOMO) of TPPS and PDI are both primarily located in delocalized *π* electrons (Figure S10, Supporting Information), which are basically in charge of the *π*–*π* interaction between TPPS and PDI.^[^ ^7a^ ^]^ Figure 1e showed the *π*–*π* stacking model of TPPS/PDI. Theoretical calculations result indicates the LUMO and HOMO of TPPS/PDI at the interface are respectively located on the PDI and TPPS (Figure 1e, left), which shows that TPPS has excellent electron‐donating properties and PDI has electron‐accepting properties (D‐A feature).^[^ ^8^, ^17^ ^]^ The theoretical calculation results show that it is easy to generate the interfacial electric field from PDI to TPPS at the interface. This interesting J–H‐type *π*–*π* accumulation model and the interface with D‐A feature can benefits spatial charge separation and serve as a fast‐transporting channel for photogenerated electrons, thereby accelerating charge mobility. Besides, the FT‐IR spectra of TPPS and PDI are shown in Figure S11 (Supporting Information).

### 2.2 D‐A Interface of TPPS/PDI Enhanced Photocatalytic Hydrogen Production

**Figure 2:**
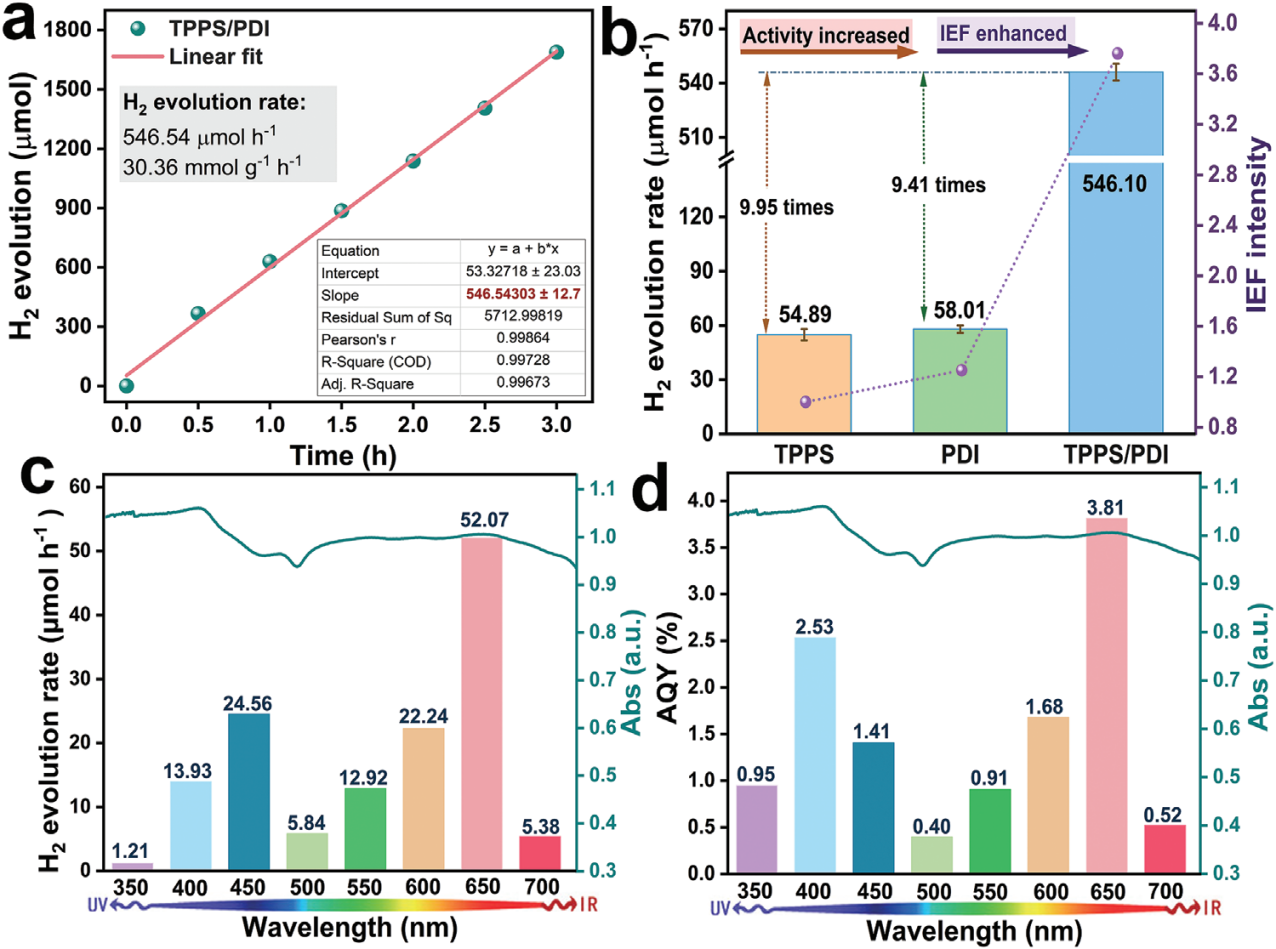
a) The photocatalytic hydrogen evolution of TPPS/PDI with time under full‐spectrum (550 mW cm^–2^). b) The comparison of internal electric field (IEF) and H_2_ evolution rate under full‐spectrum light (The mass of the catalyst is 18 mg). c) Overlayer of wavelength‐dependent hydrogen evolution and UV–vis absorption. d) Overlayer of apparent quantum yield (AQY) and UV–vis absorption.

Subsequently, the photocatalytic hydrogen production was investigated (cocatalyst: Pt, sacrificial agent: ascorbic acid). The detailed supplementary information about photocatalytic properties was in Figures S12–S17 (Supporting Information). As shown in **Figure** **2**a, the optimal H_2_ production rate of the coassembly TPPS/PDI reached up to 546.54 µmol h^–1^ under full‐spectrum light (Figure S12, Supporting Information). As exhibited in Figure 2b, the H_2_ evolution rate of TPPS/PDI was respectively 9.95 times and 9.41 times that of TPPS and PDI, which was positively associated with internal electric field (IEF). The high performance of TPPS/PDI also surpasses most of the reported porphyrin‐based materials for H_2_ evolution (Table S1, Supporting Information). It should be pointed out that the saturated H_2_ evolution rate of TPPS/PDI (546.54 µmol h^–1^) is 1.98 times and 6.27 times as high as TPPS/C_60_ (276.55 µmol h^–1^) and supramolecular zinc porphyrin (87.18 µmol h^–1^) in our previous work.^[^ ^7c,d^ ^]^ The result indicates that taking advantage of electron‐deficient property of PDI and electron‐rich characteristic of TPPS to construct the D‐A interface has greater advantages in improving photocatalytic performance. In addition, the wavelength‐dependent H_2_ production rate and the apparent quantum yield (AQY) tests of TPPS/PDI were conducted with different bandpass filters (Figure 2c,d), which were both in accord with the absorption spectrum, implying that the photoactivity was positively correlated with light absorption. The optimal H_2_ production rate (52.08 µmol h^–1^) and the maximum AQY (3.81%) both appeared at 650 nm, which attributed to excellent electron excitation at 650 nm. Here, it is important to point out that photocatalysts with such a broad spectral response are rare in the reported materials so far, which is greatly beneficial to the solar‐to‐chemical energy conversion. Besides, the solar‐to‐hydrogen conversion of TPPS/PDI was performed under AM 1.5G (Figure S16, Supporting Information), which showed that the H_2_ production rate was 57.94 µmol h^–1^. And that the photocatalytic stability of TPPS/PDI was investigated (Figure S17, Supporting Information). After testing for about 50 h, the hydrogen production of TPPS/PDI was obviously decreased, but it was still as high as 516.30 µmol h^–1^ under full‐spectrum. The decreased activity is attributable to the continued consumption of sacrifice agent. As shown in Figure S18 (Supporting Information), no obvious change was found in the XRD and IR of TPPS/PDI after long‐time photocatalytic reaction, implying a good photocatalytic stability of TPPS/PDI. Besides, as shown in Figure S19 (Supporting Information), the similar specific surface area indicates that the specific surface area has a weak effect on the photocatalytic performance.

Considering the outstanding performance of TPPS/PDI, it is necessary to study the pivotal scientific problems orienting the catalytic performance.

### 2.3 D‐A Interface of TPPS/PDI Produced Strong Interfacial Electric Field

**Figure 3:**
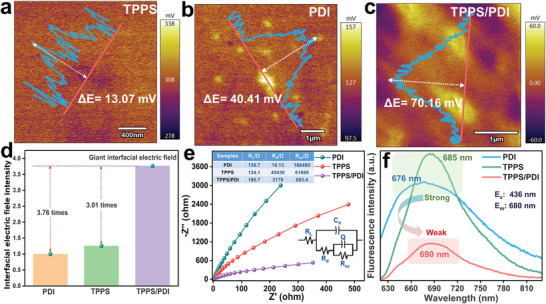
Surface potential of a) TPPS, b) PDI, and c) TPPS/PDI detected with KPFM. d) The interfacial electric field intensity of PDI, TPPS, and TPPS/PDI. e) Electrochemical impedance spectroscopy (EIS) Nyquist plots of TPPS, PDI and TPPS/PDI powder. f) Room temperature PL emission spectra of TPPS, PDI and TPPS/PDI (2 × 10^–5^ m, aqueous solution).

The interfacial electric field (IEF), as a leading kinetic factor, not only affects charge separation but also determines the direction of charge transfer. First, the strength of the interfacial electric field was explored. Research based on Zhang's group shows that the IEF enhances with the increase of surface potential and Zeta potential of the material.^[^ ^4^, ^18^ ^]^ Using the Atomic Force Microscope with Kelvin Probe is an effective method to obtain the surface potential of a given material. Clearly, the surface potential of PDI (Δ*E* = 40.41 mV) and TPPS (Δ*E* = 13.07 mV) is much lower than that of TPPS/PDI (Δ*E* = 70.16 mV) (**Figure** **3**a–c). Meanwhile, the absolute values of Zeta potential of PDI (−55.6 mV) and TPPS (−43.6 mV) are smaller than that of TPPS/PDI (−75.2 mV) (Figure S20, Table S4, Supporting Information). As a result, the coassembly TPPS/PDI exhibited a significantly stronger interfacial electric field. In order to gain insight into the IEF in TPPS/PDI, the magnitude is necessary to be evaluated. According to Lefebvre et al.,^[^ ^19^ ^]^ the surface charge density (*ρ*) and the surface voltage (*V*_s_) primarily determine the value of IEF, which can be respectively obtained by transient photocurrent density measurement and surface photovoltage spectroscopy (Figure S21, Supporting Information).^[^ ^20^ ^]^ So that in Figure 3d, TPPS/PDI showed a markedly stronger IEF, which was respectively 3.76 and 3.01 times higher than that of pure PDI and TPPS. The giant IEF would be very helpful to facilitate charge separation and transfer at the interface.

The arc radius of EIS can be used to judge the resistance of charge transfer.^[^ ^21^ ^]^ Compared with PDI and TPPS, the much smaller arc radius of TPPS/PDI (Figure 3e) indicates that the coassembly TPPS/PDI is beneficial to charge transfer. Besides, fluorescence intensity can reflect the efficiency of charge separation, because the fluorescence emission is induced by recombination of photogenerated carrier pairs.^[^ ^22^ ^]^ As shown in Figure 3f, compared with PDI and TPPS, the obvious fluorescence emission attenuation of TPPS/PDI indicates the IEF facilitates photoinduced electron transfer.

Furthermore, the interfacial charge transfer type between TPPS and PDI was explored. The energy band structures of TPPS and PDI can be determined via the UV–vis DRS and Mott–Schottky. As shown in Figure S22 (Supporting Information), the bandgaps of TPPS and PDI were respectively 1.36 and 1.56 eV, according to the Kubelka–Munk conversion function. The positive slopes of the Mott–Schottky plots in Figure S22b,c (Supporting Information) indicate that both PDI and TPPS are n‐type semiconductors. Generally speaking, for n‐type semiconductors, the flat band potential is similar to the conduction band (CB) potential.^[^ ^7e^ ^]^ Hence, the CB potential of PDI is −0.72 versus NHE (pH = 7) and the CB potential of TPPS is −1.26 V versus NHE (pH = 7). Combined with the band gap value and the CB position, the band structure diagram of TPPS and PDI is shown in Figure S22d (Supporting Information), which is the staggered energy band structures.

So here comes the question, the staggered band structures can form different charge transfer paths (type‐II or Z‐scheme), how does charge transfer? To figure it out, the work functions (WF) of TPPS and PDI were determined using in‐situ Kelvin probe force microscope (KPFM).

It is worth noting that the calibration process was carried out in argon atmosphere, which can avoid the influence of humidity and the adsorption of oxygen on the material surface. So before the contact potential difference (CPD) test of samples with KPFM, it is necessary to calibrate the work function (WF) of the probe (HQ NSC18/Pt) (Figure S23, Supporting Information). The Highly Oriented Pyrolytic Graphite (HOPG) is used as the substrate during the KPFM test. The average CPD of HOPG is 0.801 mV by the test and the work function of fresh HOPG is known as 4.600 eV. According to the formula “*V*_CPD_ = (WF_sample_ − WF_tip_)/*e*,”^[^ ^23^ ^]^ the work function of the tip is 5.401 eV. Then using the calibration probe with known work function to measure the CPD of samples, and finally calculating the work function of the sample according to the formula “*V*_CPD_ = (WF_sample_ − WF_tip_)/*e*.”

**Figure 4:**
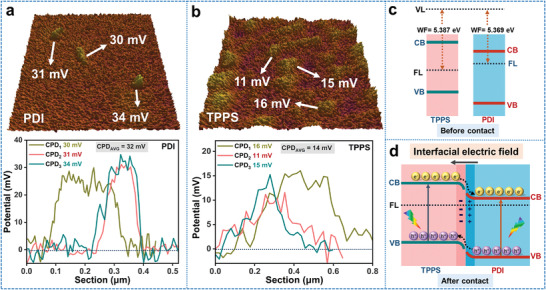
The contact potential difference (CPD) of a) PDI and b) TPPS; c) The work functions (WF) of TPPS and PDI measured using a Kelvin probe. FL, Fermi level; VL, vacuum level; CPD, contact potential differences. d) Schematic of the D‐A interface charge transfer route in the TPPS/PDI.

The results show that, the average contact potential difference (CPD) of PDI is 0.032 V and the average CPD of TPPS is 0.014 V (**Figure** **4**a,b). According to the formula *V*_CPD_ = (WF_sample_ – WF_tip_)/*e*,^[^ ^23^ ^]^ the WF of TPPS and PDI is separately 5.387 and 5.369 eV. Obviously, the Fermi level of TPPS is lower than that of PDI before contact (Figure 4c). When TPPS and PDI are in close contact, electrons are spontaneously transferred from PDI to TPPS through the interface until their Fermi levels are equal. It follows that an IEF in direction from PDI to TPPS is produced at the interface, and the energy bands of PDI and TPPS bend upward and downward respectively (Figure 4d).^[^ ^24^ ^]^ The interfacial electric field is a powerful driving force, prompting the rapid transfer of photogenerated electrons at the CB of TPPS to the CB of PDI, which strongly confirms that the charge transfer process of TPPS/PDI is type‐II process, not the Z‐scheme process. What is more, theoretical calculations indicate that the HOMO and LUMO of TPPS/PDI are respectively located on TPPS and PDI at the interface (Figure 1e), which strongly confirms again that the orientation of the interfacial electric field (IEF) at the D‐A interface is from PDI to TPPS.

### 2.4 D‐A Interface of TPPS/PDI Promoted High‐Efficiency Charge Separation

**Figure 5:**
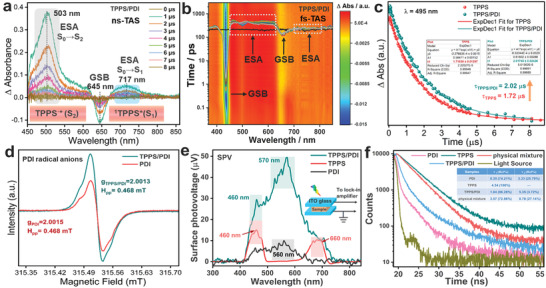
The charge‐transfer dynamics. a) Transient absorption spectra of TPPS/PDI at different time delays after nanosecond laser excitation at 440 nm (2 × 10^–5^ m, aqueous solution). b) Transient absorption spectra at different time delays after femtosecond laser excitation at 440 nm for TPPS/PDI (2 × 10^–5^ m, aqueous solution). c) Transient absorption decay kinetics of TPPS and TPPS/PDI (2 × 10^–5^ m, aqueous solution) at 495 nm. d) Room‐temperature ESR spectra under illumination. e) Surface photovoltage spectra (the schematic setup for the SPV measurements inset). f) Transient fluorescence lifetime of TPPS, PDI, and TPPS/PDI (2 × 10^–5^ m, aqueous solution).

In order to further reveal the charge carrier dynamics on the D‐A interface of TPPS/PDI, transient absorption spectra (TAS) of samples were conducted under excitation at 440 nm. Because of the excited state lifetime of PDI (at the picosecond level) is much shorter than that of TPPS (at the microsecond level), the TAS signals of TPPS/PDI primarily belong to TPPS. First, nanosecond‐TAS (ns‐TAS) was used to explore the excited state information (**Figure** **5**a, Figure S24, Supporting Information). As shown in Figure 5a, the negative ground‐state bleaching (GSB) peak of TPPS/PDI at 645 nm belongs to the Q‐band (S_0_→S_1_). And the positive excited state absorption (ESA) bands of TPPS/PDI centered at 503 nm are attributed to the Soret band of TPPS, which means S_2_ singlet state is formed, namely TPPS^•+^ (S_2_). The ESA band of TPPS/PDI centered at 717 nm belongs to the Q band (S_0_→S_1_), indicating the absorption of S_1_ singlet state ^1^TPPS* (S_1_). The nanosecond‐TAS of TPPS was illustrated in Figure S24 (Supporting Information), which is similar with the TPPS/PDI.^[^ ^7d^ ^]^ To explore the fine spectra of intermediate states, femtosecond‐TAS was further performed (Figure S25, Supporting Information). As shown in Figure 5b and Figure S25 (Supporting Information), the same GSB peaks of TPPS/PDI at 434 and 650 nm were observed, belong to transitions to the S_2_ and S_1_ excited states (B‐ and Q‐bands) of the residual monomer, respectively.^[^ ^25^ ^]^ The bi‐exciton state peak and the absorption peaks of the excited state (ESA) were also observed. However, the two GSB peaks at 490 and 712 nm were obviously disappeared, which belonged to the S_2_ and S_1_ excitonic bands of the TPPS aggregate. Combined with the fs‐TAS of PDI, the two ESA peaks of PDI aggregate started at 500 and 730 nm are located exactly at the two disappeared GSB peaks of TPPS. From the time point of view, the intensity of the absorption peaks of the individual PDI after 300 ps are not obvious. But after 300 ps, the ESA signal of TPPS/PDI did not attenuate, and there was no bleaching peak of TPPS, indicating that the excited state of TPPS and the excited state of PDI had a coupling effect on the interface. it is reasonably hypothesized that this phenomenon is caused by the generation of charge transfer state TPPS^•+^‐PDI^•–^. It needs to be emphasized that due to the long lifetime of the excited state of TPPS/PDI and the limited time window of our measurements, the dynamics of TPPS/PDI cannot be obtained from femtosecond transient absorption. However, it is obvious that TPPS/PDI has a longer lifetime of excited state than TPPS, indicating that there is an effective electron transfer between TPPS and PDI (Figure S25, Supporting Information), namely TPPS^•+^‐PDI^•–^.^[^ ^26^ ^]^

Furthermore, the lifetime of the excited state, which is crucial in photocatalytic reactions, was investigated.^[^ ^27^ ^]^ The fitting results accords with the first‐order exponential attenuation (Figure 5c). The attenuation of TPPS/PDI ( = 2.02 µs) at 495 nm is longer than that of TPPS ( = 1.72 µs), indicating that the longer‐lived charge separation state TPPS^•+^‐PDI^•–^ is formed. Therefore, the construction the D‐A interface of TPPS/PDI effectively promoted photogenerated electrons to participate in the reduction reaction.

Electron spin resonance (ESR) spectroscopy was widely used to study unpaired electrons.^[^ ^28^ ^]^ As shown in Figure 5d, the ESR signals toward PDI and TPPS/PDI both arise from the delocalization electrons, which were both smaller than free electrons ESR g value (2.0023). Obviously, the ESR intensity of TPPS/PDI was stronger than that of PDI, indicating the electron transfer from TPPS to PDI enhanced the plasma resonance effect. And consequently, more electrons with higher energy were formed, which could produce more PDI radical anion.^[^ ^29^ ^]^ The faster charge carriers transfer can also be verified by surface photovoltage (SPV) spectra. As shown in Figure 5e, the TPPS/PDI presents a stronger SPV signal, suggesting that the more efficient charge separation.^[^ ^7^, ^21^ ^]^

The time‐resolved fluorescence was used to explore the rate constants of charge separation (*k*_CS_) (Figure 5f).^[^ ^30^ ^]^ The result shows that the fluorescence attenuation of TPPS accords with the single exponent model and the fluorescence lifetime of TPPS is 4.34 ns ( = (*τ*_F_)_ref_). Obviously, the decay rate of the coassembly TPPS/PDI is much faster than that of physical mixture. The fluorescence attenuation curves of TPPS/PDI and physical mixture accord with the bi‐exponential decay function. The electron transfer from the singlet excited TPPS to PDI is corresponding to the shorter components ((*τ*_F_)_nanohybrid_) of 1.04 ns (fraction (Fr) = 96.28%) for TPPS/PDI and 3.57 ns (Fr = 72.86%) for physical mixture. And the noninteracting porphyrin entities correspond to the longer components.^[^ ^30^ ^]^ The proportion of longer components of TPPS/PDI (3.72%) is much smaller than that of physical mixture (27.14%), indicating that most of the TPPS in TPPS/PDI interact with PDI via *π*–*π* interaction. The fast decaying components serve as an additional evidence for the formation of coassembly TPPS/PDI. It is reported that the diminution of the fluorescence lifetimes can be put down to the charge separation process.^[^ ^30^ ^]^ The rate constants of charge separation (*K*_CS_) can be obtained using the shorter by the equation: *K*_CS_ = [1/*τ*(sample)] − [1/(*τ*_F_)_ref_], assuming that the quenching was due to the charge separation from the singlet excited state of TPPS. Therefore, the *K*_CS_ of TPPS/PDI can be calculated as 6.75 × 108 s^–1^, which is 13.58 times that of physical mixture (4.97 × 107 s^–1^). Such high charge separation rate of TPPS/PDI contributes to the excellent photocatalytic activity.

Based on the above analysis, the photocatalytic mechanism of TPPS/PDI is explicit. At the D‐A interface of *π*–*π* stacking, the interfacial electric field in the direction from PDI to TPPS at the interface greatly facilitates photoinduced electrons at the conduction band of TPPS transfer to the conduction band of PDI, realizing high separation efficiencies of photon‐generated carriers and more efficient hydrogen production performance.

## 3 Conclusion

A novel D‐A‐type interface between TPPS and PDI is successfully constructed for photocatalytic hydrogen production. The D‐A interface of TPPS/PDI greatly benefits the formation of the giant interfacial electric field, which dramatically promotes the charge separation and transfer. The D‐A interface of TPPS/PDI extends the lifetime of the excited state, which effectively promotes photogenerated electrons to participate in the reduction reaction. As a result, TPPS/PDI exhibits a high‐efficiency H_2_ evolution activity with outstanding rate of 546.54 µmol h^–1^ and the AQY at 650 nm achieves 3.81%, surpassing most reported organic photocatalysts. The work provides entirely new ideas for designing materials with D‐A interface to realize high photocatalytic activity.

## 4 Experimental Section

All chemicals were purchased from Sigma‐Aldrich without further refinement.

### Synthesis of Bulk PDI

The bulk perylene diimide was synthesized according to the previous work.^[^ ^31^ ^]^ First, 3‐aminopropanoic acid (28.0 mmol), imidazole (18 g) and perylene‐3,4,9,10‐tetracarboxylic dianhydride (3.5 mmol) were mixed in a three‐necked flask and heated at 120 °C for 4 h in Ar atmosphere. Then ethanol (150 mL) and hydrochloric acid (2.0 m, 300 mL) were added into the flask in succession and stirred for 24 h. And then the dark red solid was washed with deionized water until the filtrate is neutral and filtrated through a 0.22 µm filter membrane. Finally, the solid was dried at 60 °C under vacuum for 24 h. The sample was marked as bulk PDI.

### Synthesis of Self‐Assembly PDI

First, a certain quality of bulk PDI (0.1 g) was added in 100 mL deionized water. Subsequently, 200 µL triethylamine was added and then stirred for 30 min. Then 4 mL aqueous hydrochloric acid solution (4.0 m) was added and continued stirring for 6 h. Then it was washed thoroughly with distilled water until the pH of filtrate became neutral. The collected solid was dried at 60 °C under vacuum for 24 h.

### Preparation of Self‐Assembled TPPS and TPPS/PDI

First, 0.0934 g purchased TPPS and bulk PDI of different mass fractions were dispersed in deionized water (100 mL), stirred for 30 min. Then 400 µL triethylamine was added and stirred for 1 h. Then hydrochloric acid solution was added to adjust the pH to 4, and stirred at 60 °C for 6 h. At last, the solid was obtained through filtration and dried at 60 °C in a vacuum oven, marked as TPPS/PDI‐X (“X” is the mass ratio of TPPS relative to bulk PDI, namely, 0.2:1, 0.4:1, 0.6:1, 0.8:1, 1:1, and 1.2:1. Note: TPPS/PDI‐1:1 was selected for analysis, which showed the best catalytic activity).

The self‐assembly TPPS was obtained without adding bulk PDI.

## Conflict of Interest

The authors declare no conflict of interest.

## Supporting information

Supporting information

## Data Availability

The data that support the findings of this study are available from the corresponding author upon reasonable request.

## References

[1] a) H. Nishiyama , T. Yamada , M. Nakabayashi , Y. Maehara , M. Yamaguchi , Y. Kuromiya , Y. Nagatsuma , H. Tokudome , S. Akiyama , T. Watanabe , R. Narushima , S. Okunaka , N. Shibata , T. Takata , T. Hisatomi , K. Domen , Nature 2021, 598, 304; 34433207 10.1038/s41586-021-03907-3

[2] a) J. D. Xiao , H. L. Jiang , Acc. Chem. Res. 2019, 52, 356; 30571078 10.1021/acs.accounts.8b00521

[3] a) Y. Zheng , L. Lin , B. Wang , X. Wang , Angew. Chem., Int. Ed. 2015, 54, 12868; 10.1002/anie.201501788 26424620

[4] a) J. Li , L. Cai , J. Shang , Y. Yu , L. Zhang , Adv. Mater. 2016, 28, 4059; 27001143 10.1002/adma.201600301

[5] a) C. Dai , B. Liu , Energy Environ. Sci. 2020, 13, 24;

[6] a) J. Shi , R. Chen , H. Hao , C. Wang , X. Lang , Angew. Chem., Int. Ed. 2020, 59, 9088; 10.1002/anie.202000723 32162747

[7] a) Z. Zhang , X. Chen , H. Zhang , W. Liu , W. Zhu , Y. Zhu , Adv. Mater. 2020, 32, 1907746; 10.1002/adma.201907746 32596838

[8] a) Y. Chao , J. Jheng , J. Wu , K. Wu , H. Peng , M. Tsai , C. Wang , Y. Hsiao , C. Wang , C. Lin , C. Hsu , Adv. Mater. 2014, 26, 5205; 24890183 10.1002/adma.201401345

[9] a) G. B. Bodedla , W. Wong , X. Zhu , J. Mater. Chem. A 2021, 9, 20645.

[10] R. Bera , S. Chakraborty , S. K. Nayak , B. Jana , A. Patra , J. Phys. Chem. C 2019, 123, 15815.

[11] K. S. Park , K. S. Lee , J. Baek , L. Lee , B. H. Son , Y.‐E. Koo Lee , Y. H. Ahn , W. I. Park , Y. Kang , M. M. Sung , Angew. Chem., Int. Ed. 2016, 55, 10273. 10.1002/anie.201603961 27461905

[12] a) L. Zhao , R. Ma , J. Li , Y. Li , Y. An , L. Shi , Biomacromolecules 2008, 9, 2601; 18700742 10.1021/bm8004808

[13] a) F. Würthner , Chem. Commun. 2004, 1564; 10.1039/b401630k 15263926

[14] Y. Sheng , W. Li , Y. Zhu , L. Zhang , Appl. Catal., B 2021, 298, 120585.

[15] a) S. M. Safar Sajadi , S. Khoee , Sci. Rep. 2021, 11, 2832; 33531578 10.1038/s41598-021-82256-7 PMC7854723

[16] a) B. Z. Wang , S. S. Zheng , A. Saha , L. P. Bao , X. Lu , D. M. Guldi , J. Am. Chem. Soc. 2017, 139, 10578; 28686429 10.1021/jacs.7b06162

[17] a) M. Wolf , J. I. T. Costa , M. B. Minameyer , T. Drewello , A. C. Tomé , D. M. Guldi , J. Phys. Chem. C 2019, 123, 28093;

[18] J. Li , G. Zhan , Y. Yu , L. Zhang , Nat. Commun. 2016, 7, 11480. 27157679 10.1038/ncomms11480 PMC4865814

[19] a) P. Lefebvre , J. Allègre , B. Gil , H. Mathieu , N. Grandjean , M. Leroux , J. Massies , P. Bigenwald , Phys. Rev. B 1999, 59, 15363;

[20] F. L.e Formal , K. Sivula , M. Grätzel , J. Phys. Chem. C. 2012, 116, 26707.

[21] G. Wang , Q. Sun , Y. Liu , B. Huang , Y. Dai , X. Zhang , X. Qin , Chem. ‐ Eur. J. 2015, 21, 2364. 25487284 10.1002/chem.201405047

[22] S. Usai , S. Obregón , A. I. Becerro , G. Colón , J. Phys. Chem. C 2013, 117, 24479.

[23] a) D. Zhao , Y. Wang , C.‐L. Dong , Y.‐C. Huang , J. Chen , F. Xue , S. Shen , L. Guo , Nat. Energy 2021, 6, 388;

[24] a) F. Liu , R. Shi , Z. Wang , Y. Weng , C.‐M. Che , Y. Chen , Angew. Chem., Int. Ed. 2019, 58, 11791; 10.1002/anie.201906416 31241810

[25] E. Collini , C. Ferrante , R. Bozio , J. Phys. Chem. C 2007, 111, 18636.

[26] K. Ohkubo , H. Kotani , J. Shao , Z. Ou , K. M. Kadish , G. Li , R. K. Pandey , M. Fujitsuka , O. Ito , H. Imahori , S. Fukuzumi , Angew. Chem., Int. Ed. 2004, 116, 871. 10.1002/anie.200352870 14767957

[27] a) S. Fukuzumi , K. Ohkubo , Dalton Trans. 2013, 42, 15846; 24141827 10.1039/c3dt51883c

[28] a) M. Fedin , E. Bagryanskaya , H. Matsuoka , S. Yamauchi , S. L. Veber , K. Maryunina , E. Tretyakov , V. Ovcharenko , R. Sagdeev , J. Am. Chem. Soc. 2012, 134, 16319; 22963168 10.1021/ja306467e

[29] W. Wei , Z. Wei , D. Liu , Y. Zhu , Appl. Catal., B 2018, 230, 49.

[30] S. K. Das , N. K. Subbaiyan , F. D'Souza , A. S. D. Sandanayaka , T. Hasobe , O. Ito , Energy Environ. Sci. 2011, 4, 707.

[31] J. Wang , W. Shi , D. Liu , Z. Zhang , Y. Zhu , D. Wang , Appl. Catal., B 2017, 202, 289.

