# Supplementary material for: Electron Donor–Acceptor Interface of TPPS/PDI Boosting Charge Transfer for Efficient Photocatalytic Hydrogen Evolution
Source: Adv Sci (Weinh). 2022 Apr 11;9(17):2201134. doi: 10.1002/advs.202201134 (PMC9189676; doi:10.1002/advs.202201134)
Supplement: Supplementary file 1 — Supporting information [file ADVS-9-2201134-s001.pdf]

ADVANCED SCIENCE
Open Access

Supporting Information

for *Adv. Sci.*, DOI 10.1002/advs.202201134

Electron Donor–Acceptor Interface of TPPS/PDI Boosting Charge Transfer for Efficient Photocatalytic Hydrogen Evolution

*Jun Yang, Jianfang Jing, Wenlu Li and Yongfa Zhu**

ADVANCED SCIENCE

Supporting Information

Electron Donor-Acceptor Interface of TPPS/PDI Boosting Charge Transfer for Efficient Photocatalytic Hydrogen Evolution

*Jun Yang, Jianfang Jing, Wenlu Li and Yongfa Zhu**

Dr. J. Yang, Dr. J. Jing, Dr. W. Li and Prof. Y. Zhu

Department of Chemistry, Tsinghua University, Beijing, 100084, P. R. China

E-mail: zhuyf@tsinghua.edu.cn

Table of Contents

1. Experimental details 3
- 1.1 Characterizations 3
- 1.2 Photoelectrochemical tests 3
- 1.3 Transient absorption spectra 4
- 1.4 Calculation of theoretical solar spectrum efficiency 4
- 1.5 Photocatalytic hydrogen evolution experiments 5

2. Results and discussion 6
- 2.1 Supplementary information for the structure of materials 6
- 2.2 Supplementary information about photocatalytic properties 12
- 2.3 Supplementary information for strong interfacial electric field 18
- 2.4 Supplementary information for electron transfer dynamics 23

3. References 25

1. Experimental details

1.1 Characterizations

The HT 7700 transmission electron microscope (TEM, Hitachi, Japan) was used to investigate the morphology of samples, and the acceleration voltage of the electron beam was 100 kV. The JEOL JEM-2010F instrument was used as the high-power transmission electron microscope (HRTEM) and the acceleration voltage of the electron beam was 200 kV. X-ray diffraction (XRD) patterns of the samples were obtained on a Rigaku D/max-2400 X-ray diffractometer using a Cu Kα1 (λ = 0.15418 nm) at 40 kV and 200 mA, with a scan step of 0.02°. The infrared spectrum was tested on the VERTEX 70 Fourier Transform Infrared Spectrometer (Bruker, Germany). The ultraviolet-visible diffuse reflectance spectrum (UV-Vis) was obtained by the U-3010 spectrometer (Hitachi, Japan). The steady-state fluorescence spectrum test was recorded on the Edinburgh FS5 photoluminescence spectrometer with an excitation wavelength of 400 nm. Time-resolved photoluminescence spectra were collected on Edinburgh FLSP920 fluorescence spectrometer with an excitation wavelength of 400 nm and the detection wavelength is at 670 nm. The samples were ultrasonically dissolved in deionized water at a concentration of 2×10^{-5} M. The atomic force microscopystudy was carried out using Cypher VRS with Kelvin probe (HQ NSC18/Pt). The surface photovoltage (SPV) measurements were conducted with a home-built instrument as previously reported. [1] Monochromatic light resource was a 500 W xenon lamp (CHF XQ500W) with a double-prism monochromator (Omni-λ 3005). The slit width was set at 3 mm. The photovoltage signal was amplified by a lock-in amplifier (SR830-DSP) with a light chopper (SR540). The resolution of spectrum was 1 nm. The raw surface photovoltage data were normalized using the illuminometer (Zolix UOM-1S). Zeta potential was measured with a Horiba SZ-100 Nano Particle analyzer with the temperature of the holder being 25.0 ℃. BET surface area measurements were recorded by N_2 adsorption at 77 K using a Micrometrics (ASAP 2010V5.02H) surface area analyzer.

1.2 Photoelectrochemical tests

The photoelectrochemical tests was performed on the CHI660B electrochemical workstation (Shanghai Chenhua Instrument Company). The electrochemical impedance spectroscopy (EIS), Mott-Schottky and photocurrent tests were carried on a three-electrode electrochemical workstation with a working electrode, a Pt wire counter electrode, and a saturated calomel reference electrode. Firstly, 5 mg of the prepared sample was

added to 2 ml of deionized water, and a uniform suspension was formed after ultrasound for 30 minutes. The suspension was dropped dropwise on the FTO glass sheet (2 cm x 4 cm), and then dried at room temperature for about 24 hours, and then the FTO glass slide was dried at 80°C for about 24 hours. A 300 W xenon lamp was used as the light source and 0.1 M Na_2SO_4 solution was used as the electrolyte solution.

1.3 Transient absorption spectra

The nanosecond transient absorption spectra were obtained by a laser source of the homemade spectra setup (Spectra Physics Company, American). The output pulse was split into two beams. The first beam was used to generate 440 nm laser pulses. The second beam with weaker energy was focused on a CaF_2 plate to generate a white light continuum as the probe (Energetiq company, EQ-99). The time delay between the pump beam and the probe pulses was controlled by a motorized delay stage. The samples were ultrasonically dissolved in deionized water at a concentration of 2×10^{-5} M, and nitrogen gas was bubbled for 30 minutes. The samples were ultrasonically dissolved in deionized water, and nitrogen gas was bubbled for 30 minutes.

Furthermore, to explore the fine spectra of intermediate states, femtosecond- transient absorption spectrometer was further performed. [2]

1.4 Calculation of theoretical solar spectrum efficiency

The spectrum efficiency gives an indicator for evaluating the spectral utilization of materials. The theoretical solar spectrum efficiency was calculated with as-collected solar spectrum and UV-VIS-NIR absorption spectrum of TPPS/PDI. The solar spectrum with collected with Optic Fiber Spectrometer, AULTPP-P4000, CEAULIGHT. The UV-VIS-NIR absorption spectrum was collected on Cary 5000, Varian. And the theoretical spectrun efficiency equals to the the wavelength range of the material absorption spectrum divided by the measured solar spectrum. It should be pointed out that the absorption range of the material needs to be counted from the intrinsic absorption of the electronic transition and ended in the smaller wavelength in solar spectrum or absorption spectrum. So, for TPPS/PDI, it should be 853-300 nm. For the measured solar spectrum, it is 1068-300 nm. Thus, the therotical spectrum efficiency can be calculated as follow equation.[4]

$$\text{Therotical spectrum efficiency} = \frac{Absorption\ range}{Measured\ solar\ spectrum\ range} = \frac{853 - 300\ nm}{1068 - 300\ nm} \approx 72\%$$

1.5 Photocatalytic hydrogen evolution experiments

The photocatalytic performance of samples was evaluated by the performance of H_2 production under visible light and full-spectrum light on a glass closed gas system (Labsolar-6A, Beijing Perfectlight Technology Co., Ltd., **Figure S1**). The photocatalyst powder was ultrasonically dispersed in 100 mL deionized water. Pt was used as co-catalyst via in-situ photodeposition on the surface of samples and 0.2 mol L^{-1} ascorbic acid (AA) as the sacrificial agent. A 300 W xenon lamp was used as the light source (Full spectrum, light intensity 600 mW cm^{-2}). The production of H_2 was detected by an online gas chromatograph (TCD detector, Ar carrier, 5Å molecular sieve column) at given times intervals. The H_2 evolution test with loading different amount of Pt and (c) the H_2 evolution with different sample weight were studied. [3]

The apparent quantum yield (AQY) at various monochromatic lights (350 nm, 400 nm, 450 nm, 500 nm, 550 nm, 600 nm, 650nm and 700 nm) were measured in the aid of different band-pass filters (FWHM=15 nm). The irradiation area is 1 cm^{-2} (300 W xenon lamp, PerfectLight). The average intensity was determined by an optical power meter (S310C connected to the PM100D console, Thorlabs). 18 mg photocatalyst was used. The AQY was calculated as following equation: [3]

$$\mathrm{AQY} = \frac{2 \times the\ number\ of\ evolved\ H_2\ molecules}{the\ number\ of\ incident\ photons} \times 100\%$$

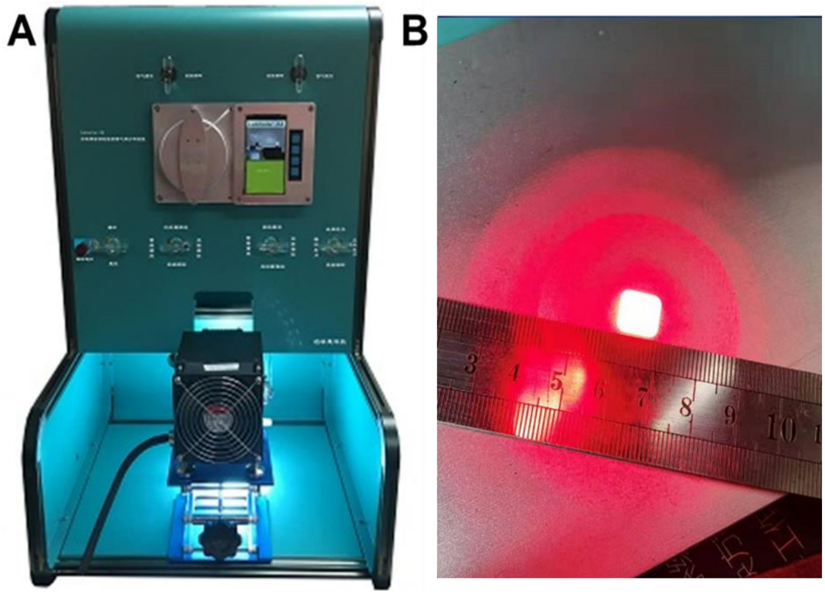

Figure S1. Photograph of the photocatalytic on-line analytical system (Labsolar-6A, Beijing Perfectlight Technology Co., Ltd.); (b) The light spot with the area of 1 cm^2 for AQY test (Here, take λ=650 nm for example).

2. Results and discussion

2.1 Supplementary information for the structure of materials

The morphology and highly crystalline structure of samples was shown in **Figure S2**. The transmission electron microscopy (TEM) images (**Figure Sa1**, **S2a2, Sa3**) showed TPPS, PDI and TPPS/PDI all mainly exhibited nanowires structure via π-π stacking by dissolution-precipitation process. It is observed that TPPS and TPPS/PDI exhibit high crystallinity. Obviously, d-spacing of 0.327 nm and 0.263 nm can be observed, corresponding to PDI and TPPS, respectively. The structural advantage of high crystallinity is conducive to the construction of strong interfacial electric field between TPPS and PDI, which makes it have good charge separation and transfer capabilities. [4]

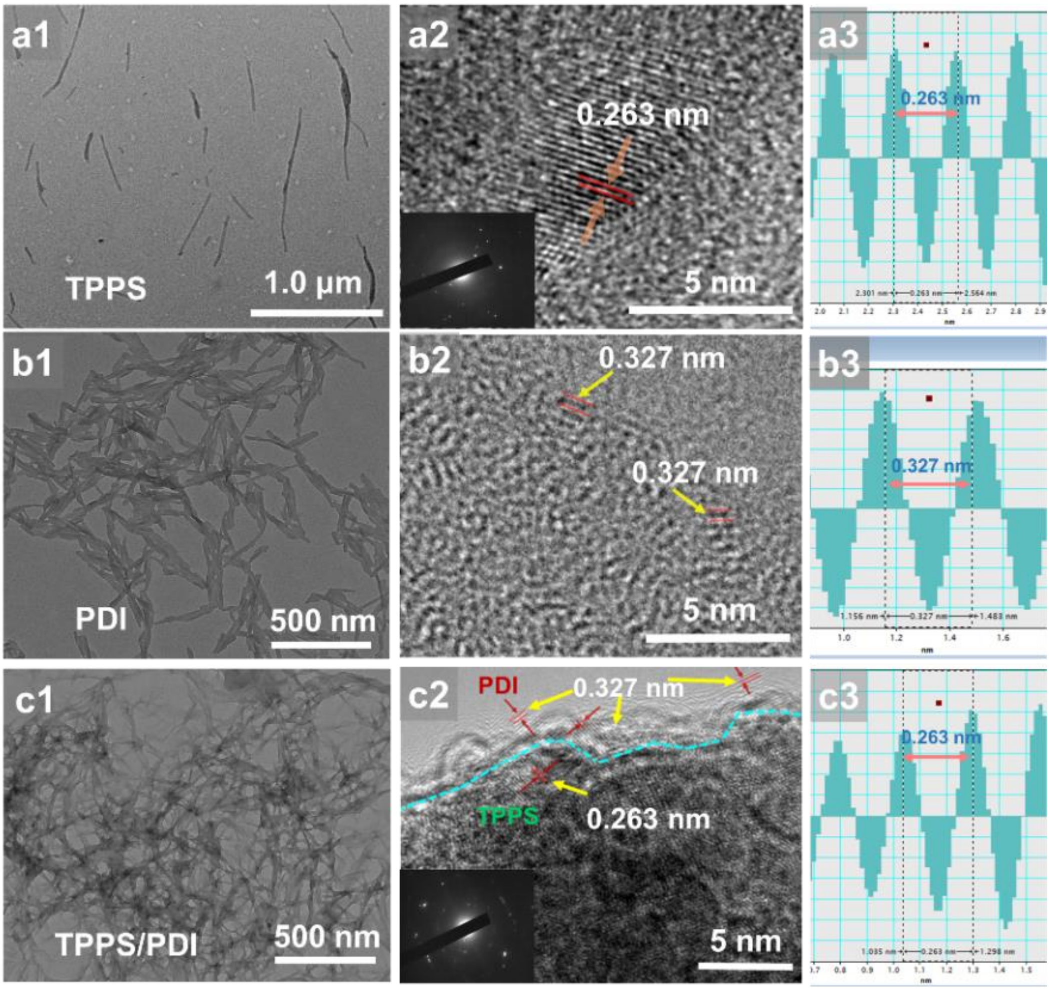

Figure S2. TEM and HRTEM images of TPPS (a1, a2, a3), PDI (b1, b2, b3) and TPPS/PDI (c1, c2, c3).

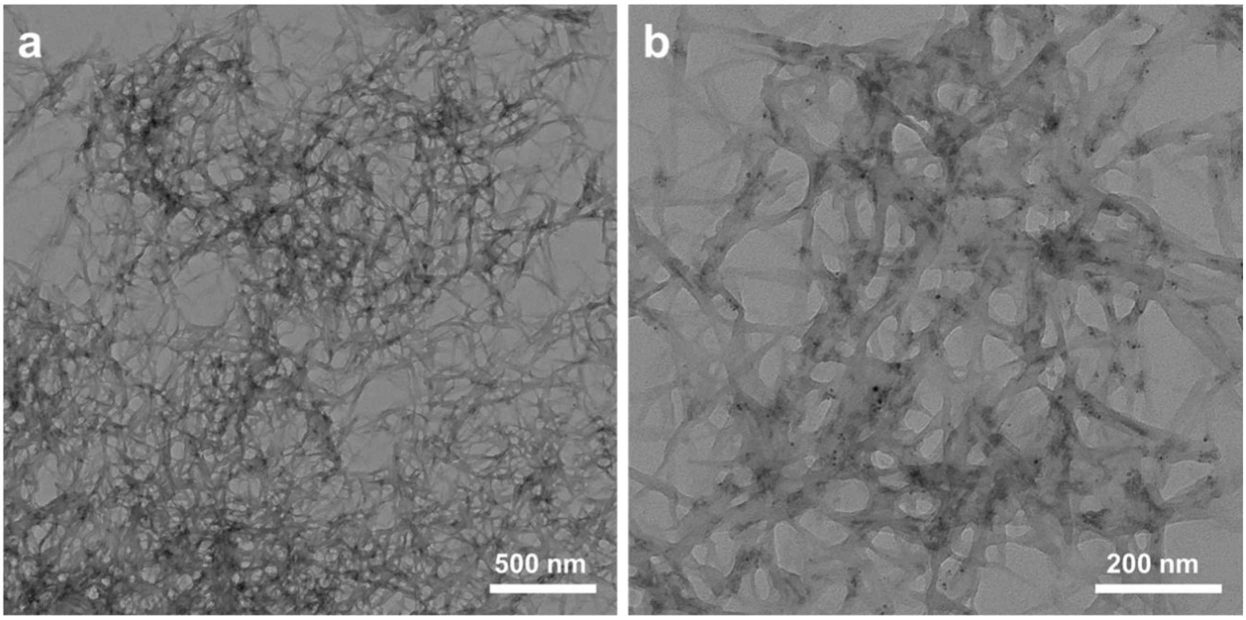

Figure S3. TEM spectra of TPPS/PDI at different magnifications.

For clarity, **figure S3** shows TEM maps of TPPS/ PDI at different magnifications. The results show that PDI is stacked with long-range H-type π-π stacking as nanofibers, while TPPS are mostly distributed on PDI nanofibers with short-range J-type π-π stacking as nano-dots.

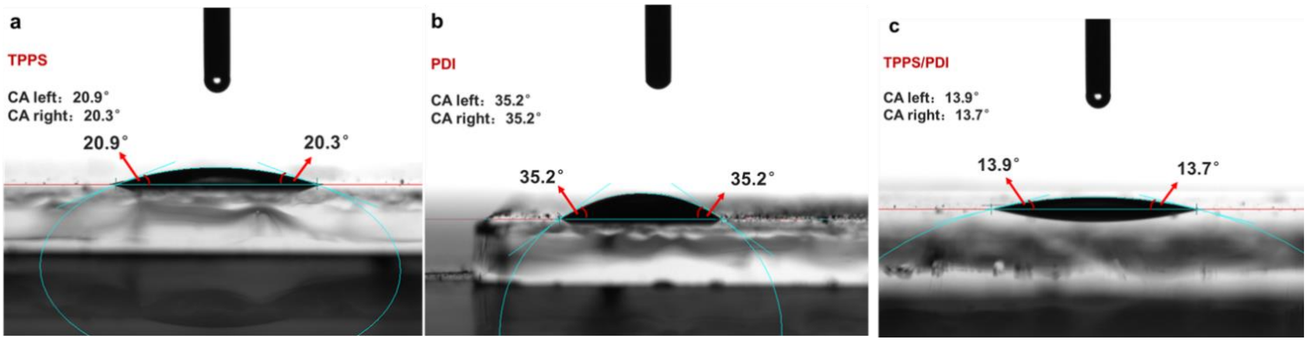

Figure S4. The contact Angles of (a) TPPS, (b) PDI and (c) TPPS/PDI.

The results of contact angles indicate TPPS, PDI and TPPS/PDI have an excellent hydrophilicity due to the existence of carboxylic and sulfonic groups, which is favorable for catalytic reaction.

As shown in **Figure S5**, the UV-vis spectra show that the typical Soret band and Q bands of porphyrin. Simultaneously, the Soret band of TPPS exhibited red shift and the number of Q-bands reduced compared with the TPPS monomer, indicating the J-type accumulation is formed. [5]

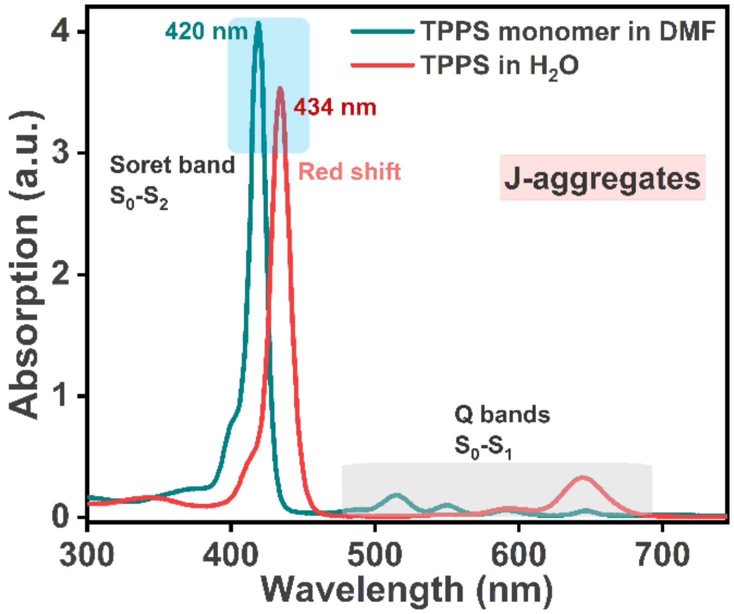

Figure S5. Comparison of UV–vis absorption spectrums for TPPS dissolved in DMF and TPPS dispersed in H_2O

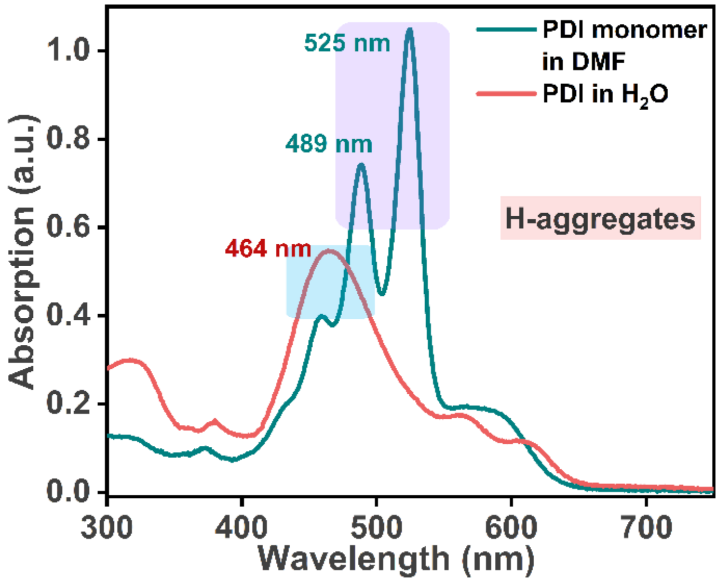

Figure S6. Comparison of UV–vis absorption spectrums for PDI dissolved in DMF and PDI dispersed in H_2O

The UV–vis absorption spectra of PDI monomer and PDI nanofiber gel were shown in **Figure S6**. The absorption spectrum of PDI dissolved in DMF shows three pronounced peaks in the range of 400–650 nm, which correspond to the 0-0, 0–1 and 0–2 electronic transitions of monomeric PDI molecules, respectively. [6] After self-assembly, the maximum absorption has a great blue-shift and the fine curve structure of absorption spectrum was lost, indicating the strong H-type stacking between the PDI skeletons in supramolecular nanofibers. [7]

The UV–vis absorption spectra of TPPS/PDI was shown in **Figure S7**, from which it can be clearly concluded that TPPS and PDI show J-type and H-type accumulation respectively. As for the co-assembly TPPS/PDI, the short-range J-type accumulation of TPPS can better imitate the chlorophyll in photosynthesis in nature. And the long-range H-type stacking of PDI can help provide better long-range electrons delocalization, which was benefit to the migration of charge carriers. [8]

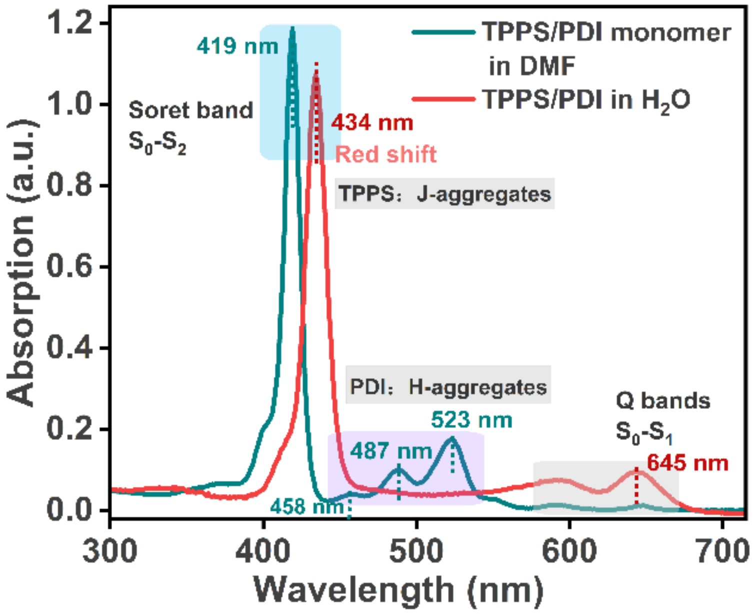

Figure S7. Comparison of UV–vis absorption spectrums for TPPS/PDI dissolved in DMF and TPPS/PDI dispersed in H_2O

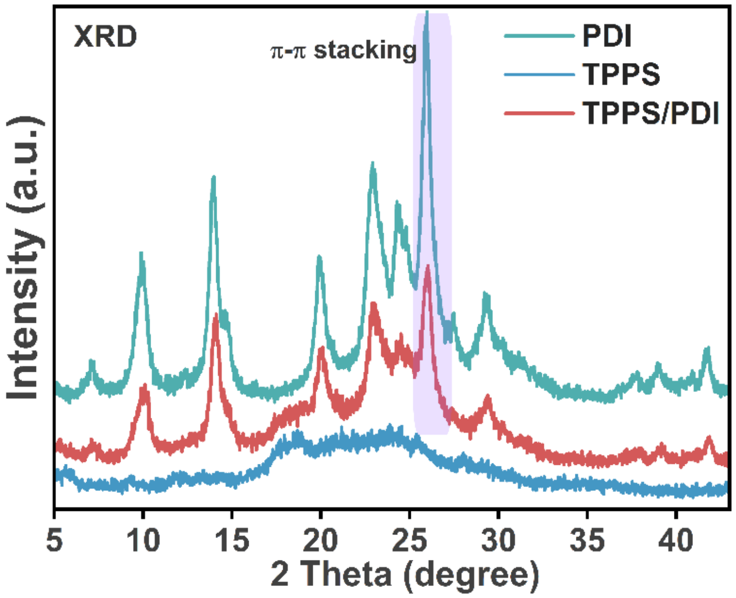

Figure S8. Powder XRD pattern of PDI, TPPS and TPPS/PDI heterostructure

The XRD patterns in **Figure S8** showed the typical d-spacing of π-π stacking characteristic peak. [9]

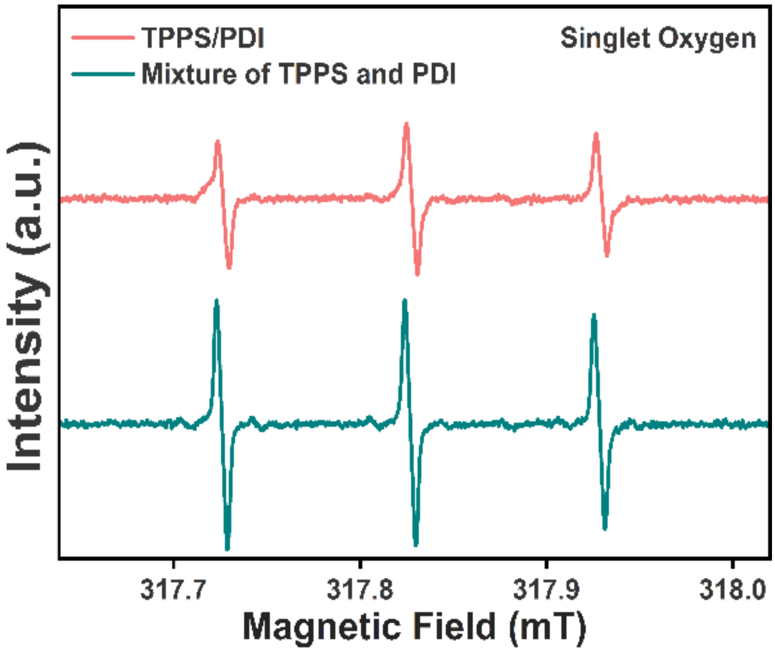

Figure S9. ESR signals of singlet oxygen ($^1O_2$) under light irradiation for TPPS/THPP heterostructure and physical mixture of TPPS and PDI (2,2,6,6-tetramethylpiperidine (TEMP) as capture agent)

The strong π–π interaction between PDI and TPPS can be revealed by electron spin resonance (ESR) technique (**Figure S9**). TPPS/PDI exhibited a significant decrease in singlet oxygen generation compared with physical mixture of TPPS and PDI, implying that π–π stacking between TPPS and PDI resulted in aggregation-induced quenching of the excitation energy, thereby decreasing the singlet oxygen signals. [10]

Based on theoretical calculation results, the highest occupied molecular orbital (HOMO) and lowest unoccupied molecular orbital (LUMO) of both TPPS and THPP molecule are mainly distributed in delocalized π electrons (**Figure S10**), and these frontier orbitals are mainly responsible for π–π interaction between TPPS and PDI. Theoretical calculations were carried out through Density Functional Theory (DFT) with Gaussian 09 software. B3LYP/6-31+G(d) method was used for molecular optimization. The highest occupied molecular orbital (HOMO), lowest unoccupied molecular orbital (LUMO) and orbital distribution were analyzed and drawn using Multiwfn program [11] and VMD software. [12]

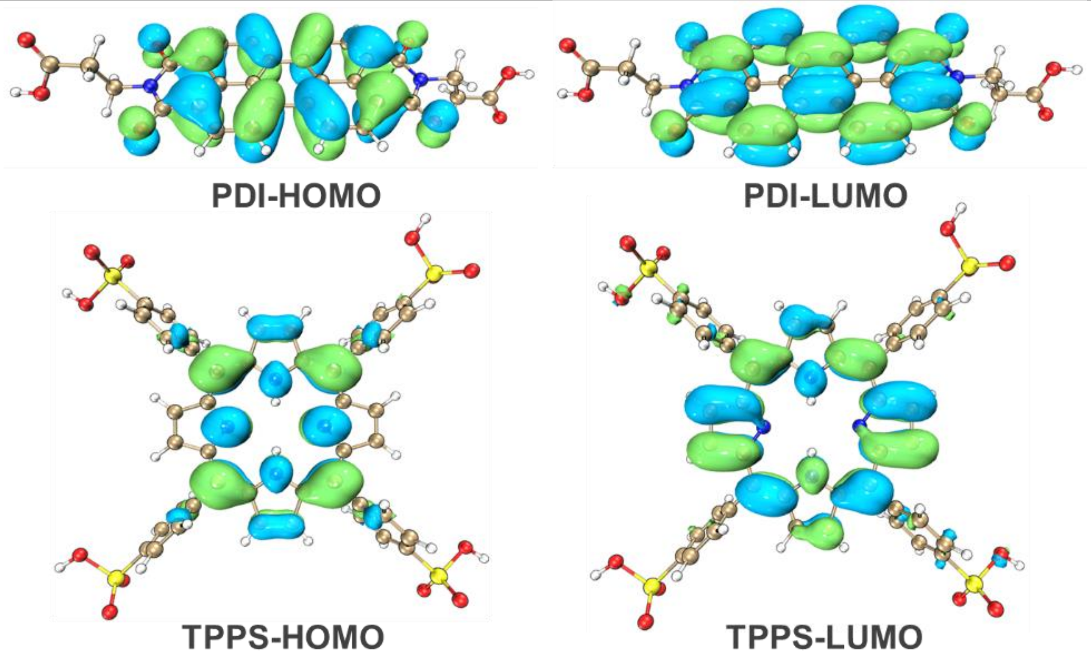

Figure S10. Diagram of the frontier molecular orbitals by DFT method

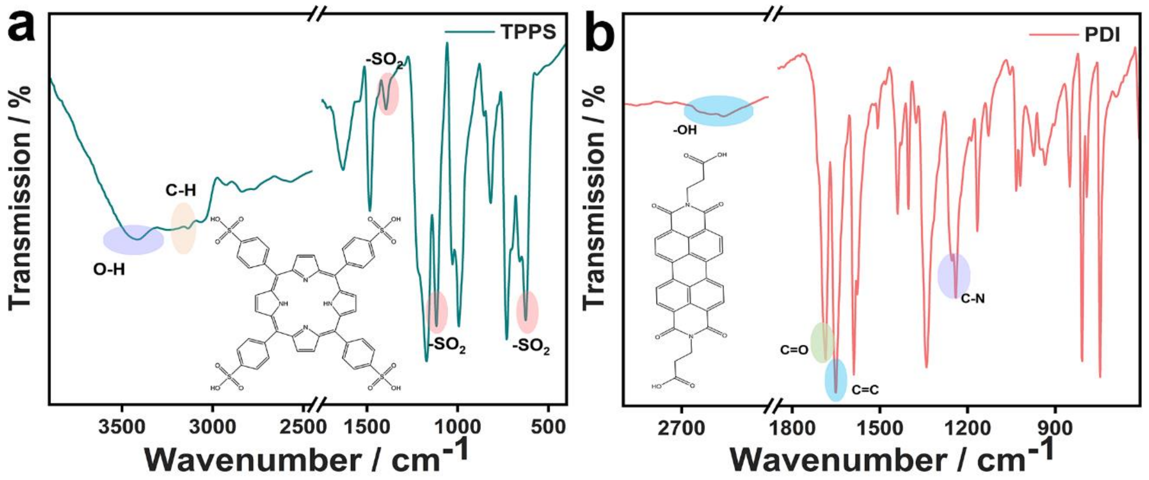

Figure S11. FT-IR spectra of (a) TPPS and (b) PDI.

As shown in **Figure S11a**, TPPS show wide adsorption bands at 3600~2500 cm^{-1}. The characteristic peak of TPPS at about 3400 cm^{-1} is attributed to the –OH stretching vibration of the sulfonic acid group, and the 3124 cm^{-1} is attributed to the C-H stretching vibration absorption peak on the benzene ring. The strong absorption peaks of 1117 cm^{-1} and 1390 cm^{-1} correspond to the symmetric and asymmetric stretching vibrations of $-SO_2$, respectively. The scissor vibration and rocking vibration of $-SO_2$ can be represented by 570±60 cm^{-1} and 520±40 cm^{-1}. [13] The IR-spectrum of PDI as shown in **Figure S11b** shows that the functional group –OH, C=O, C=C and C-N.

2.2 Supplementary information about photocatalytic properties

As shown in **Table S1**, compared with recently reported porphyrin-based materials, TPPS/PDI is the highest catalysts in terms of hydrogen production performance among these materials.

Table S1 Comparison of different porphyrin-based materials

Catalysts	Light Source	Sacrificial agents	Cocatalyst	H_2 (μmol h^{-1})	Ref.
TPPS/PDI	Full spectrum	Ascorbic acid	Pt	**546.54**	**This work**
TPPS/PDI	λ > 420 nm	Ascorbic acid	Pt	**525.18**	**This work**
TPPS/C_{60}	Full spectrum	Ascorbic acid	Pt	276.55	[3]
TPPS/C_{60}	λ > 420 nm	Ascorbic acid	Pt	272.25	[3]
ZnTCPP	Full spectrum	Ascorbic acid	Pt	87.18	[14]
ZnTCPP	λ > 420 nm	Ascorbic acid	Pt	74.91	[14]
USTC-8(In)	λ > 380 nm	Triethylamine	Pt	3.41	[15]
mTCPP-CN	λ > 400 nm	EDTA	Pt	54.3	[16]
HNTM	λ > 400 nm	Triethylamine	Pt, Ir	10.1	[17]
Al-TCPP	λ > 380 nm	Triethanolamine	Pt, Ru	0.65	[18]
SA-MNS	λ > 420 nm	Ascorbic acid	Pt	56.6	[19]
InTPP	λ > 400 nm	Ascorbic acid	Pt	0.85	[20]
ZnTPyP	λ > 420 nm	Ascorbic acid	Pt	235.5	[21]
THPP	λ > 420 nm	Ascorbic acid	Pt	78	[22]

Note：According to the article '*Angew. Chem. Int. Ed. 2020, 59, 18312–18320*', it is not appropriate to compare performance numbers only without considering the test conditions, because light source, catalyst quality, amount of supported co-catalyst, sacrificial agent concentration and so on all affect catalytic performance. But compared with TPPS and PDI alone, by constructing the D-A interface, a very significant improvement in catalytic performance is indeed obtained under the same test conditions. The rapid electron transfer at the D-A interface plays a key role in the improvement of catalyst performance.

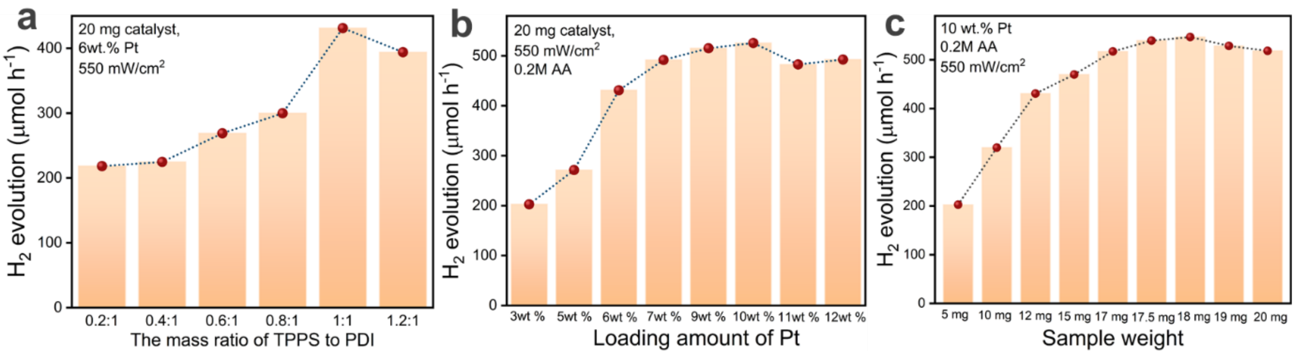

Figure S12. (a) The photocatalytic hydrogen evolution rate of TPPS/PDI-X with time under full-spectrum. ("X" represents the feeding mass fraction of TPPS relative to bulk PDI, namely 0.2:1, 0.4:1, 0.6:1, 0.8:1, 1:1, 1.2:1.) (b) The H_2 evolution with loading different amount of Pt; (c) The H_2 evolution with different sample weight. Note：All catalytic reactions were performed under light for 3 h, and the gas was detected by gas chromatography every 30 minutes.

The photocatalytic hydrogen evolution of TPPS/PDI-X with different mass ratio were studied ("X" represents the feeding mass fraction of TPPS relative to bulk PDI, namely 0.2:1, 0.4:1, 0.6:1, 0.8:1, 1:1, 1.2:1). As shown in **Figure S12a**, the catalytic performance of TPPS/PDI gradually improved with the increase of TPPS content at the beginning. When the feeding mass fraction of TPPS relative to PDI is 50%, TPPS/PDI showed the best H_2 evolution activity. However, when the mass of TPPS continues to increase, the performance of TPPS/PDI begins to decline slightly. It is mainly because excessive TPPS will prevent the interface between TPPS and PDI from absorbing sufficient light. There is a balance between charge separation and light absorption, where TPPS/PDI-50% may represent the balance point. Moderate amounts of PDI can enhance the separation of photon-generated carriers, resulting in an increased catalytic activity. Combined with Figure S12b and Figure S12c, the optimal catalytic conditions can be obtained as follows: The feeding mass fraction of TPPS relative to PDI is 50%, the mass fraction of platinum is 10% and the mass of catalyst is 18 mg.

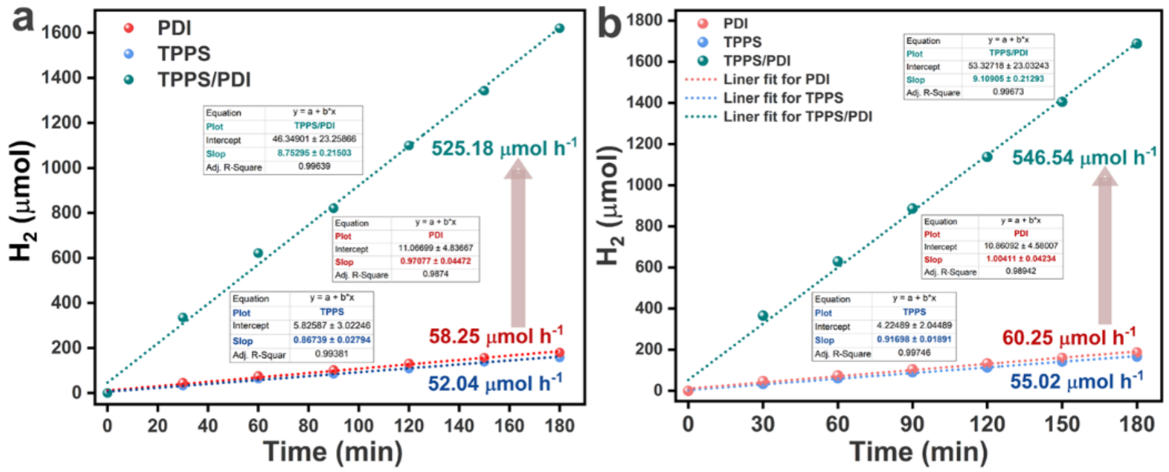

Figure S13. The photocatalytic hydrogen evolution with TPPS and TPPS/PDI (a) under visible light and (b) under full spectrum.

The amount of evolved hydrogen of TPPS and TPPS/PDI with time under visible light and full spectrum is respectively shown in **Figure S13a** and **Figure S13b**. The result shows that the photocatalytic of the TPPS/PDI system is much higher than that of pure PDI and TPPS. The H_2 production process conformed to the pseudo-first-order kinetics.

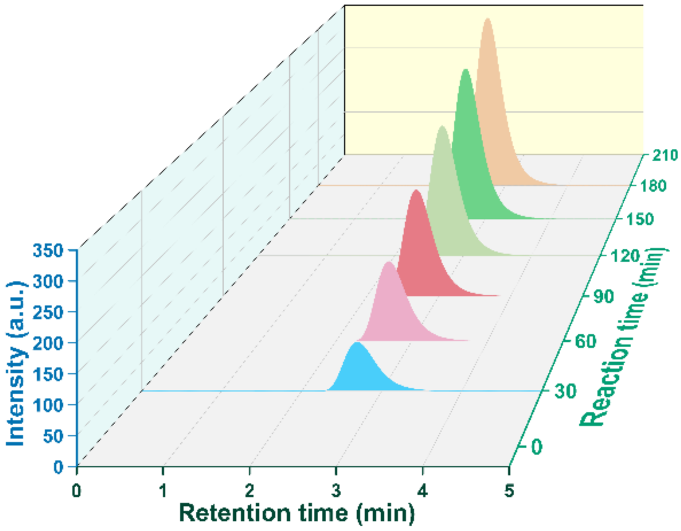

Figure S14. The time-dependent gas chromatogram of H_2 evolution of TPPS/PDI under full-spectrum irradiation.

As shown in **Figure S14**, the peak of hydrogen (Retention Time =2.646 min) increased obviously with the prolonger reaction time. The GC map peak area are listed as **Table S2**. The quantitative coefficient of the peak area in our GC is 0.000249 (μmol per unit area, **Figure S15**), and the mass of photocatalyst was 18 mg. From the fitting table, the reaction rate (slope) of TPPS/PDI is 546.54 μmol h^{-1} (30.36 mmol g^{-1} h^{-1}), R^2 =0.99746 (**Figure 2a**).

Table S2. The GC map peak area and the amount of H_2 evolution

Reaction time (min)	Area 1	Hydrogen amount μmol	Hydrogen amount per unit weight of photocatalyst (18 mg) mmol g^{-1}
0	0	0	0
30	1469292	365.85	20.33
60	2526220	629.03	34.95
90	3558919	886.17	49.23
120	4568551	1137.57	63.20
150	5643933	1405.34	78.07
180	6779238	1688.03	93.78

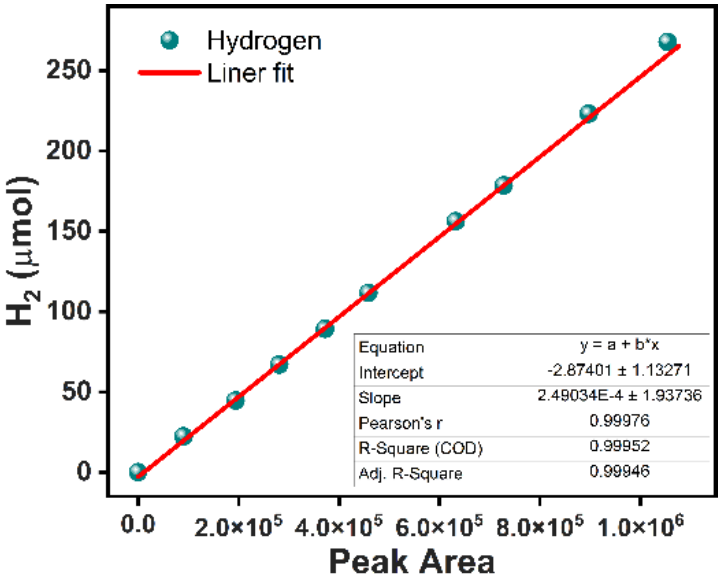

Figure S15. The calibration curves of photocatalytic H_2 evolution.

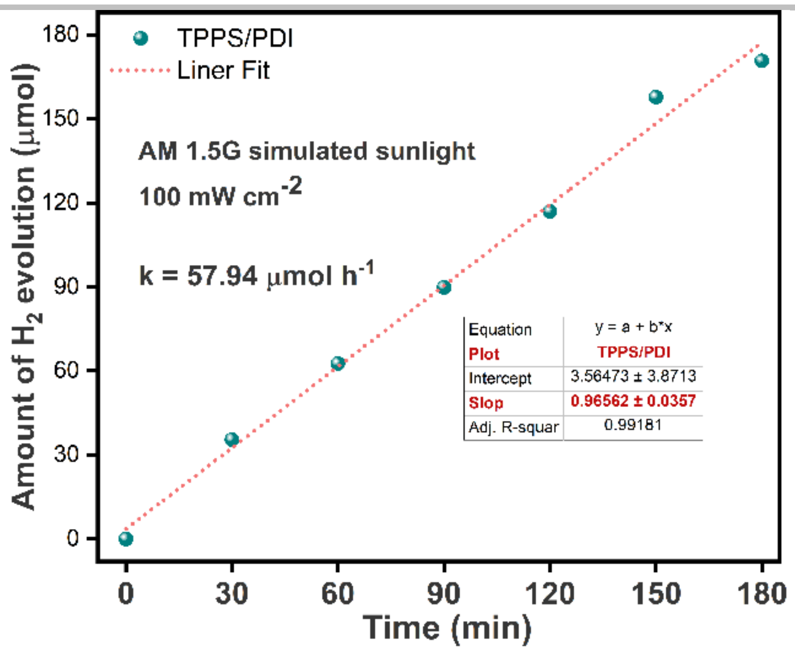

Figure S16. The time-dependent H_2 evolution under AM 1.5G simulated sunlight of TPPS/PDI.

The solar-to-hydrogen (STH) energy-conversion efficiency was examined under AM 1.5G simulated sunlight, as shown in **Figure S16**. After 3 h of light irradiation, the H_2 evolution rate achieved 57.94 μmol h^{-1}. Since the ascorbic acid was used as sacrificial agent in H_2 evolution reaction over TPPS/PDI, it is difficult to obtain the Gibbs energy of the reaction accurately, so an accurate STH value cannot be given.

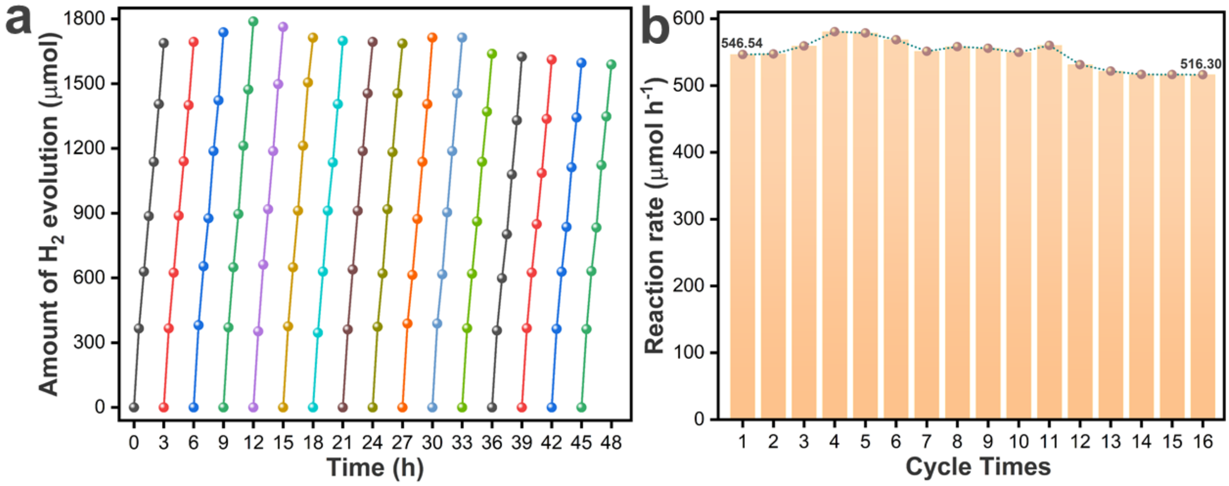

Figure S17. (a) The cyclic photocatalytic hydrogen evolution of TPPS/PDI with time under full-spectrum. (b) The corresponding H_2 evolution rate of TPPS/PDI.

As shown in **Figure S17**, after testing for about fifty hours, the hydrogen production of TPPS/PDI was obviously decreased, but still as high as 516.30 μmol h^{-1} (28.68 mmol $g^{-1}h^{-1}$) under full-spectrum. The decreased activity is attributable to the continued consumption of sacrifice agent.

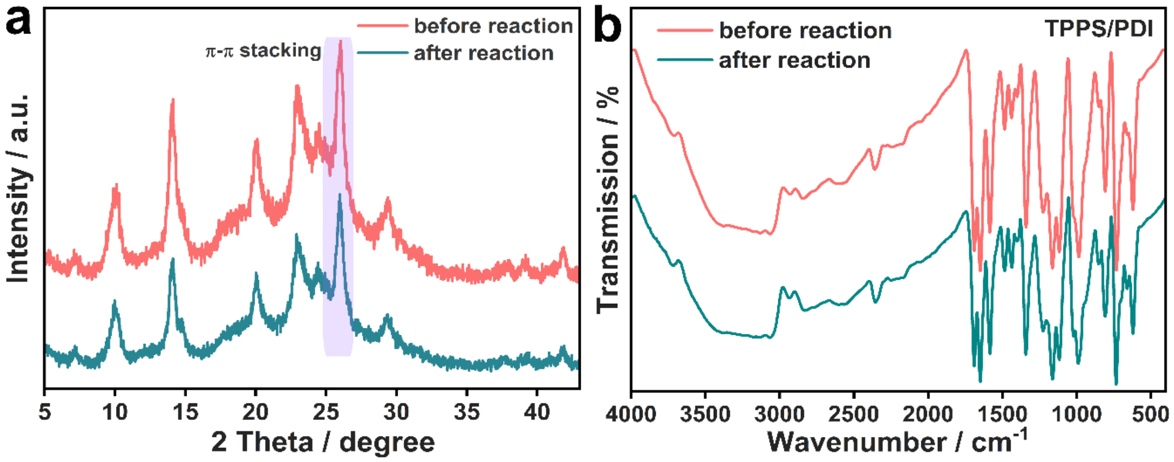

Figure S18. The XRD and IR spectra of TPPS/PDI before and after photocatalytic reaction

As shown in **Figure S18**, no obvious change was found in the XRD and IR of TPPS/PDI after long-time photocatalytic reaction, implying a good photocatalytic stability of TPPS/PDI.

Table S3. Wavelength dependent AQY of photocatalytic H_2 evolution over TPPS/PDI.[a]

Wavelength (nm)	350	400	450	500	550	600	650	700
H_2 evolution (μmol)	0.23	0.61	0.95	0.54	1.48	2.61	7.47	0.87
Light intensity (mW/cm^2)	2.27	2.01	4.97	9.05	9.89	8.62	10.02	7.95
Irradiation area (cm^2)	1.00	1.00	1.00	1.00	1.00	1.00	1.00	1.00
Irradiation time (h)	2.00	2.00	2.00	2.00	2.00	2.00	2.00	2.00
AQY (%)	0.95	2.53	1.41	0.40	0.91	1.68	3.81	0.52

[a] Condition: 10wt.% Pt as co-catalysts; Ascorbic acid (0.2 mol L^{-1}, 100 mL) as sacrificial agent; A fiber source equipped with various band-pass filters as the light source.

The specific calculation method takes 650 nm as an example.

λ =650 nm：

The number of incident photons:

$$\mathrm{N} = \frac{E\lambda}{hc} = \frac{10.02 \times 1.00 \times 10^{-3} \times 2 \times 3600 \times 650 \times 10^{-9}}{6.626 \times 10^{-34} \times 3 \times 10^{8}} = 2.36 \times 10^{20}$$

AQY：

$$\mathrm{AQY} = \frac{2 \times the\ number\ of\ evolved\ H_2\ molecules}{N} \times 100\% = \frac{2 \times 6.02 \times 10^{23} \times 7.47 \times 10^{-6}}{2.36 \times 10^{20}} \times 100\% = 3.81\%$$

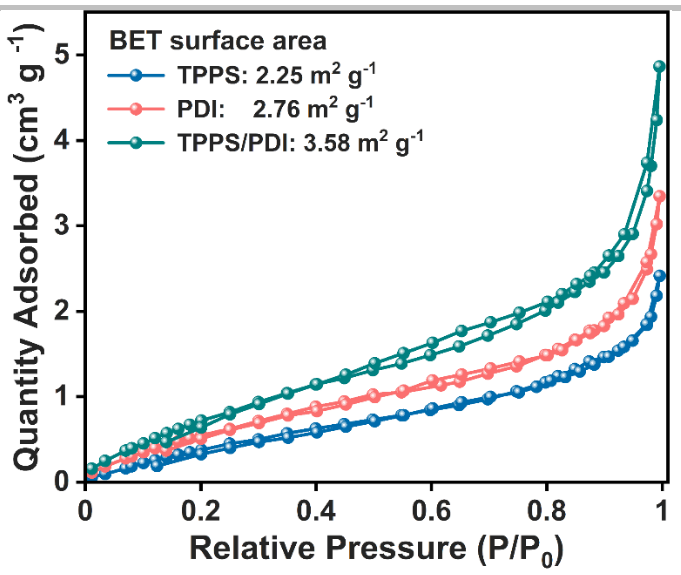

Figure S19. N_2 adsorption-desorption isotherms of TPPS, PDI and TPPS/PDI

The N_2 adsorption-desorption measurements were further conducted to explore the specific surface area of catalysts. As shown in **Figure S19**, the specific surface areas of TPPS, PDI and TPPS/PDI are 2.25 m^2 g^{-1}, 2.76 m^2 g^{-1} and 3.58 m^2 g^{-1}, respectively. The similar specific surface area indicates that the specific surface area has a weak effect on the photocatalytic performance.

2.3 Supplementary information for strong interfacial electric field

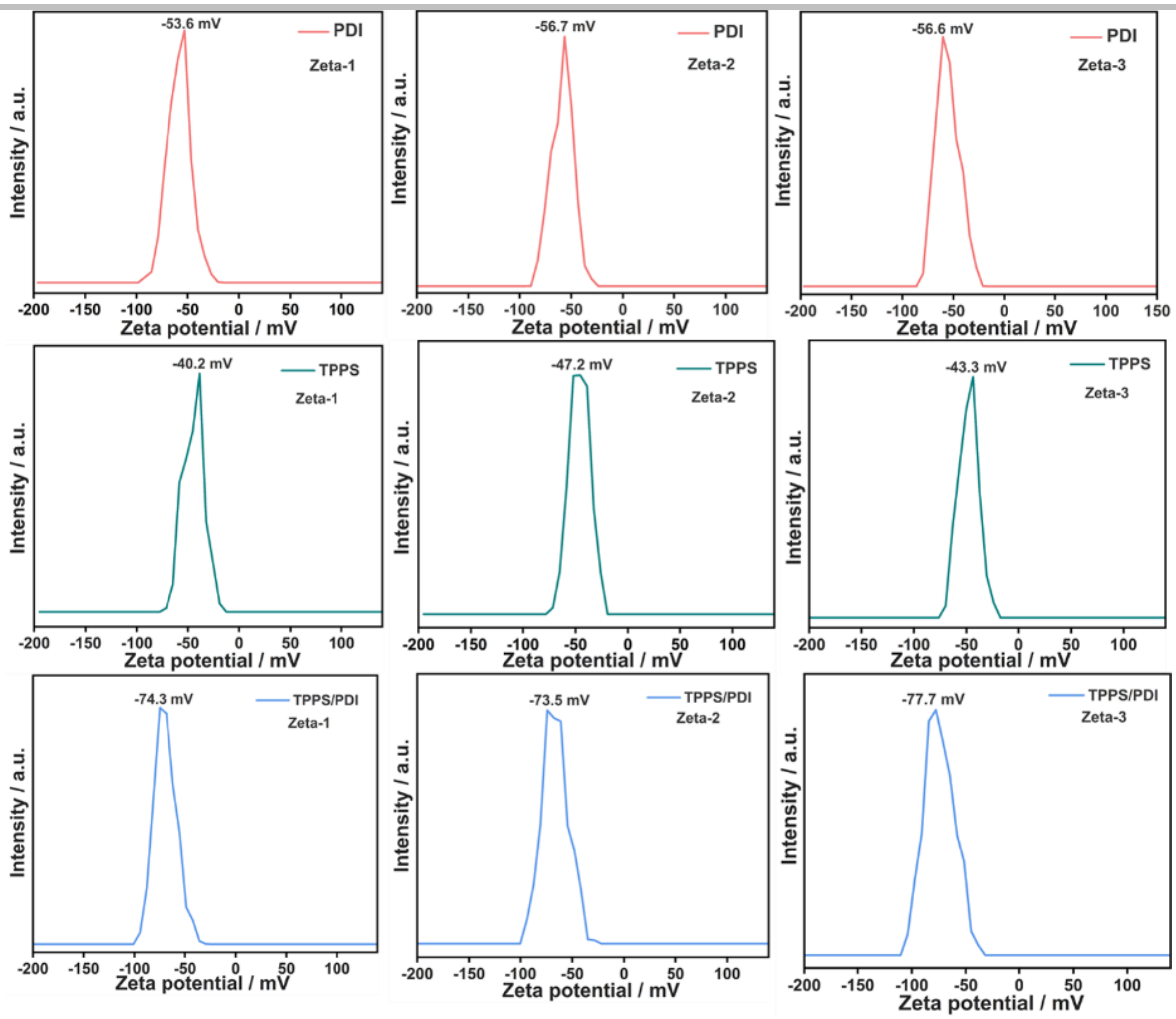

Figure S20. Zeta potential of PDI, TPPS and TPPS/PDI

Table S4. The Zeta potential of samples (mV)

The test sequence	PDI / mV	TPPS / mV	TPPS/PDI / mV
1	-53.6	-40.2	-74.3
2	-56.7	-47.2	-73.5
3	-56.6	-43.3	-77.7
Average	-55.6	-43.6	-75.2

As shown in **Figure S20** and **Table S4**, PDI, TPPS and TPPS/PDI are all negatively charged. The average zeta potential ξ value of TPPS/PDI is negative 75.2 mV, whose absolute value is larger than that of PDI (-55.6mV) and TPPS (-43.6 mV).

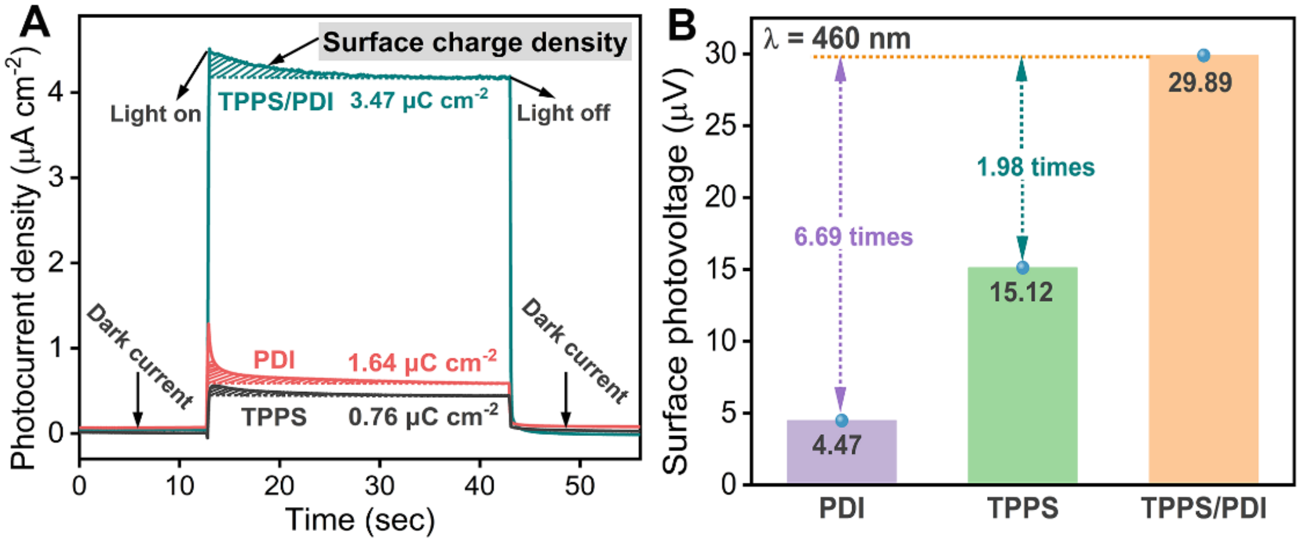

Figure S21. (A) The surface charge density and (B) surface photovoltage intensity of PDI, TPPS and TPPS/PDI.

The surface charge density of PDI, TPPS and TPPS/PDI is shown in **Figure S21A**. According to Le Formal and Gratzel et al., the value is proportional to the number of positive charges accumulated at the surface, by integrating the measured transient photocurrent density minus the steady-state values of photocurrent with respect to time.[23] The surface photovoltage was acquired by measuring their surface photovoltage spectrum (**Figure 5e**, at λ=460 nm). The result shows that both the surface charge density and surface photovoltage intensity of TPPS/PDI are higher than those of PDI and TPPS, which indicates that the built-in electric field of TPPS/PDI is higher than that of the individual PDI and TPPS according to the following equation developed by Lefebvre et al. [24]

$$F_s=(-2V_s\rho/\varepsilon\varepsilon_0)^{1/2}$$

Where Fs is the internal electric field magnitude, Vs is the surface voltage, ρ is the surface charge density, Ɛ is the low-frequency dielectric constant, and ε_0 is the permittivity of free space. The above equation reveals that the internal electric field magnitude is mainly determined by the surface voltage and the charge density because Ɛ and ε_0 are two constants. It can be found that the internal electric field intensity of TPPS/PDI is 3.76 times and 3.01 times higher than that of pure TPPS and PDI, respectively.

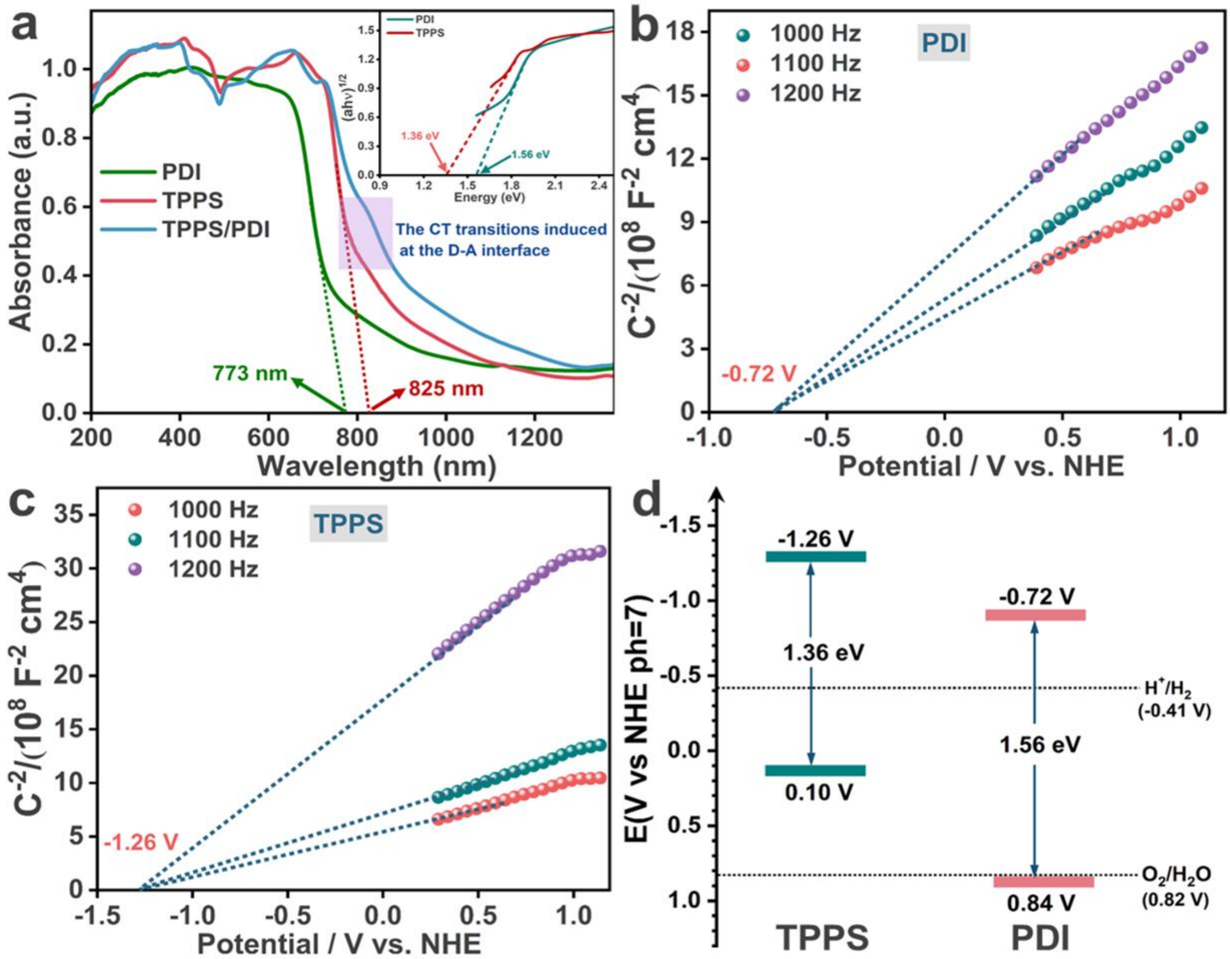

Figure S22. (a) UV–vis diffuse reflection spectroscopy of samples (The inset is the corresponding Tauc plots of samples), (b) Mott-Schottky plots of PDI, (c) Mott-Schottky plots of TPPS and (d) The schematic drawing of redox potentials of TPPS and PDI.

As shown in **Figure S22a**, the band gap of samples can be obtained by UV–vis diffuse reflection spectroscopy and the corresponding Tauc plots. The band gap of PDI and TPPS is 1.56 eV and 1.36 eV, respectively. In the Mott-Schottky plots (**Figure S22b** and **Figure S22c**), the positive slopes demonstrate the n-type semiconductor characteristic of TPPS and PDI. In general, for an n-type semiconductor, the flat-band potential is approximately at the conduction band potential. [25] Thus, as shown in **Figure S22b**, the conduction band potential of -0.72 V vs. NHE (pH=7) for PDI can be obtained, and its valence band potential be calculated to 0.84 V vs. NHE (pH=7). The conduction band potential of -1.26 V vs. NHE (pH=7) for TPPS can be obtained from its Mott-Schottky plots (**Figure S22c**), and its valence band potential be calculated to 0.10 V vs. NHE (pH=7). The schematic drawing of redox potentials of TPPS and PDI is conclusively shown in **Figure S22d**.

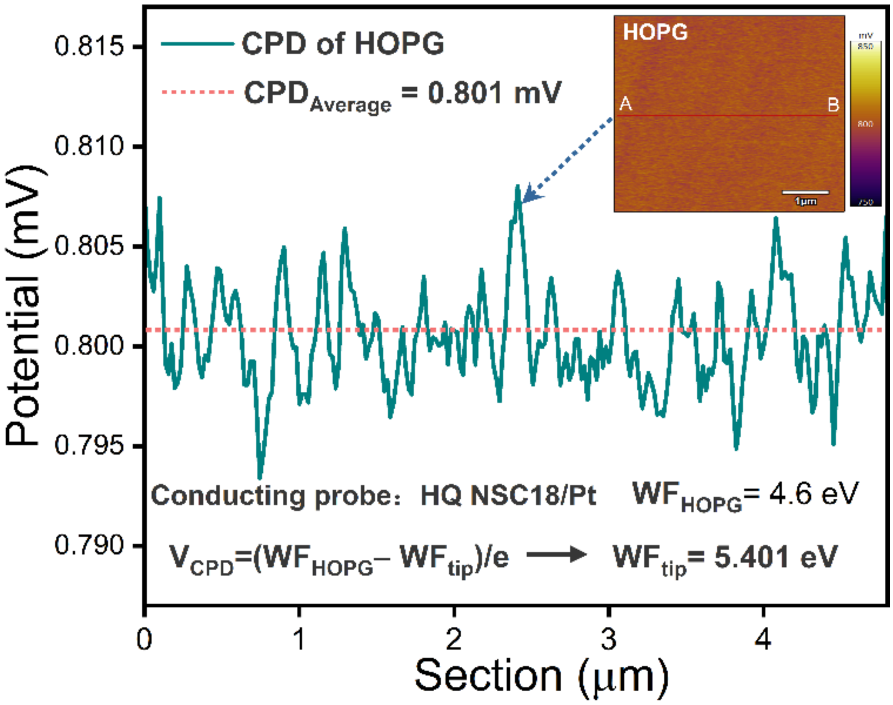

Figure S23. The contact potential difference (CPD) of the substrate HOPG

Before the contact potential difference (CPD) test of samples with KPFM, it is necessary to calibrate the work function (WF) of the probe (HQ NSC18/Pt). The Highly Oriented Pyrolytic Graphite (HOPG) is used as the substrate during the KPFM test. The average CPD of HOPG is 0.801 mV by the test and the work function of fresh HOPG is known as 4.600 eV (**Figure S23**). According to the formula "V_{CPD}=(WF_{sample}-WF_{tip})/e", the work function of the tip is 5.401 eV. Then using the calibration probe with known work function to measure the CPD of samples, and finally calculating the work function of the sample according to the formula "V_{CPD}=(WF_{sample}-WF_{tip})/e". [26] It's worth noting that the calibration process was carried out in argon atmosphere, which can avoid the influence of humidity and the adsorption of oxygen on the material surface.

The CPD of samples of the different zones of samples was shown in Figure 4 and Table S5. The average contact potential difference (CPD) between PDI and probe is 0.032 V and the average CPD between TPPS and probe is 0.014 V, thus the WF of TPPS and PDI can be calculated as 5.387 eV and 5.369 eV, respectively, according to the formula "V_{CPD}=(WF_{sample}-WF_{tip})/e". [26]

Table S5. The contact potential difference (CPD) of samples

The different zones of samples	PDI / mV	TPPS / mV
1	31	11
2	30	15
3	34	16
**Average**	**32**	**14**

2.4 Supplementary information for electron transfer dynamics

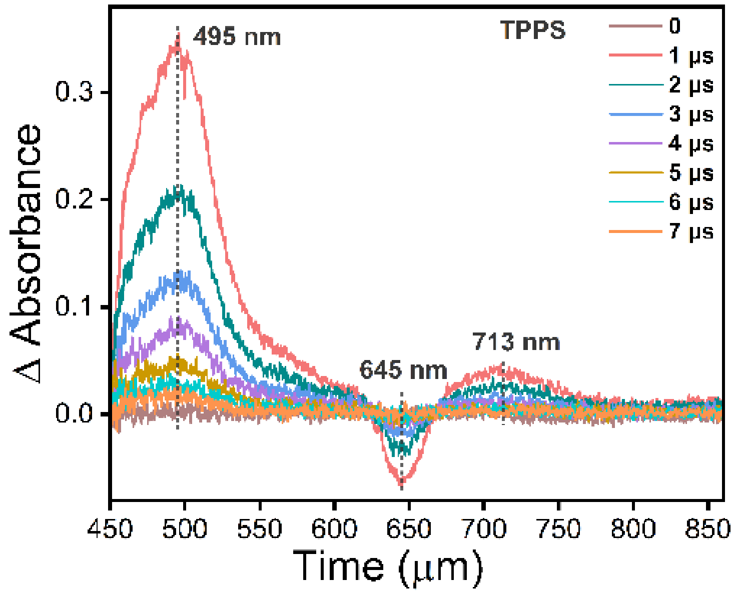

Figure S24. Nanosecond transient absorption spectra (ns-TAS) of TPPS at different time delays after nanosecond laser excitation at 440 nm.

As shown in **Figure S24**, TPPS has an obvious negative bleaching peak near the wavelength of 645 nm, and two positive excitation absorption bands near 495 nm and 713 nm. The bleaching peak near 645 nm is located near the Q-band ($S_0 \rightarrow S_1$). The transient absorption band between 450 nm and 610 nm coincides with the Soret band ($S_0 \rightarrow S_2$) of TPPS, indicating the formation of S_2 singlet state, namely $TPPS^{\bullet+}$ (S_2). The absorption at near 716 nm is located in the Q band ($S_0 \rightarrow S_1$), which belongs to the absorption of S_1 singlet state, namely, $^1TPPS^*$ (S_1). [3]

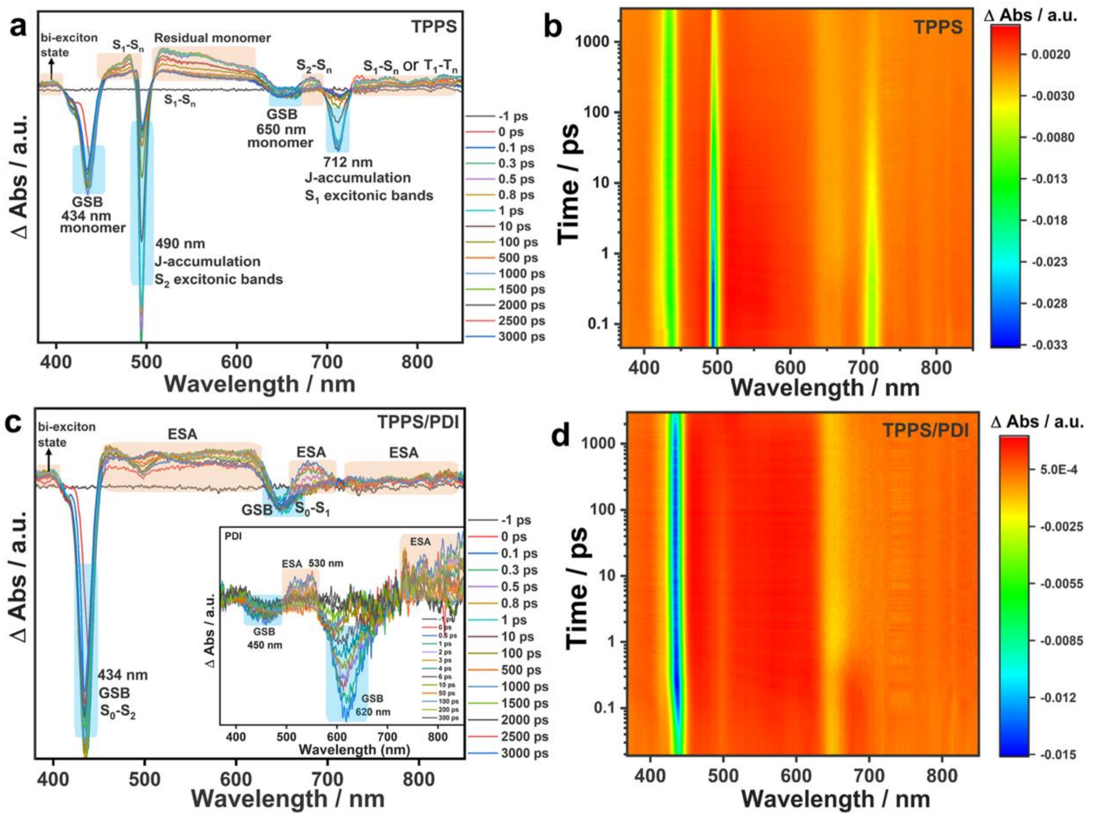

Figure S25. (a) 1D-fs-TAS of TPPS, (b) 3D-fs-TAS of TPPS, (c) 1D-fs-TAS of TPPS/PDI (the inset is the fs-TAS of PDI), (d) 3D-fs-TAS of TPPS/PDI, in deionized water (pH=3) at room temperature obtained upon femtosecond pump probe experiments following 440 nm laser excitation.

To explore the fine spectra of intermediate states, the femtosecond transient absorption (fs-TAS) in the visible region was further performed as shown in **Figure S25**. It should be noted that due to the excited state lifetime of PDI (at the picosecond level) is much shorter than that of TPPS (at the microsecond level), the TAS signals of TPPS/PDI are mainly attributed to TPPS. As shown in **Figure S25a** and **S25b**, the negative ground-state bleaching (GSB) peaks and the positive excited state absorption (ESA) bands of TPPS aggregation are obviously presented. The spectra almost agree with the results reported by Collini et al. [27] According to the literature, the GSB peaks at about 434 nm and 650 nm belong to transitions to the S_2 and S_1 excited states (B- and Q-bands) of the residual monomer, respectively. And the other two bleaching signals at 490 and 712 nm, relative to the S_2 and S_1 excitonic bands of the TPPS aggregate, respectively. [27] Two broad ESA peaks centered at about 480 nm and between 500 nm and 630 nm were also recorded, which were typical feature observed in many porphyrins, [28] and it can be associated mainly with excited-state absorption (ESA) from both S_2 and S_1 states up to higher singlet energy states (S_n). It shows a positive amplitude just between 660 and 700 nm, indicating that there are excited states S_n that

can be reached from S_2 through a one-photon absorption process. [27] A very weak ESA is also present between 730 and 850 nm. Some authors observed the same feature for similar porphyrins and attribute it either to S1→Sn transitions or to triplet-triplet excited-state transitions. Besides, the band observed at 400 nm of the J-aggregate can be attributed to a two-photon resonant state. [27] **Figure S25b** visually shows the distribution of transient spectra of self-assembly TPPS.

As shown in **Figure S25c** and **Figure S25d**, the same GSB peaks of TPPS/PDI at 434 nm and 650 nm were observed, belong to transitions to the S_2 and S_1 excited states (B- and Q-bands) of the residual monomer, respectively. The bi-exciton state peak and the absorption peaks of the excited state (ESA) were also observed. However, the two GSB peaks at 490 nm and 712 nm were obviously disappeared, which belonged to the S_2 and S_1 excitonic bands of the TPPS aggregate. Combined with the fs-TAS of PDI, the two ESA peaks of PDI aggregate started at 500 nm and 730 nm are located exactly at the two disappeared GSB peaks of TPPS. From the time point of view, the intensity of the absorption peaks of the individual PDI after 300 ps are not obvious. But after 300 ps, the ESA signal of TPPS/PDI did not attenuate, and there was no bleaching peak of TPPS, indicating that the excited state of TPPS and the excited state of PDI had a coupling effect. it is resonalbly hypothesized that this phenomenon is caused by the generation of charge transfer state $TPPS^{\bullet+}$-$PDI^{\bullet-}$. It needs to be emphasized that due to the long lifetime of the exciton state of TPPS/PDI and the limited time window of our measurements, the dynamics of TPPS/PDI cannot be obtained from femtosecond transient absorption. However, it is obvious that TPPS/PDI has a longer exciton state than TPPS, indicating that there is an effective electron transfer between TPPS and PDI.

3. References

[1] X. Chen, Y. Xu, X. Ma, Y. Zhu, *Nat. Sci. Rev.* **2020**, *7*, 652.

[2] F. Liu, R. Shi, Z. Wang, Y. Weng, C.-M. Che, Y. Chen, *Angew. Chem. Int. Edit.* **2019**, *58*, 11791.

[3] J. Yang, J. Jing, Y. Zhu, *Adv. Mater.* **2021**, *33*, 2101026.

[4] Z. Zhang, Y. Zhu, X. Chen, H. Zhang, J. Wang, *Adv. Mater.* **2019**, *31*, 1806626.

[5] a) L. Zhao, R. Ma, J. Li, Y. Li, Y. An, L. Shi, *Biomacromolecules* **2008**, *9*, 2601; b) J. Li, K. Ueno, H. Uehara, J. Guo, T. Oshikiri, H. Misawa, *J. Phys. Chem. Lett.* **2016**, *7*, 2786; c) K.L. Gurunatha, E. Dujardin, *J. Phys. Chem. C* **2013**, *117*, 3489.

[6] F. Würthner, *Chem. Commun.* **2004**, 1564.

[7] Z. Chen, V. Stepanenko, V. Dehm, P. Prins, L.D.A. Siebbeles, J. Seibt, P. Marquetand, V. Engel, F. Würthner,

Chem. Eur. J. **2007**, *13*, 436.

[8] a) R. Bera, S. Chakraborty, S. K. Nayak, B. Jana, A. Patra, *J. Phys. Chem. C* **2019**, *123*, 15815; b) D. Liu, J. Wang, X. Bai, R. Zong, Y. Zhu, *Adv. Mater.* **2016**, *28*, 7284.

[9] Y. Sheng, W. Li, Y. Zhu, L. Zhang, *Appl. Catal. B Environ.* **2021**, *298*, 120585.

[10] J. Wang, D. Liu, Y. Zhu, S. Zhou, S. Guan, *Appl. Catal. B: Environ*, **2018**, *231*, 251.

[11] Tian Lu, Feiwu Chen, *J. Comput. Chem.* **2012**, *33*, 580.

[12] a) W. Humphrey, A. Dalke, K. Schulten, *Journal of Molecular Graphics* **1996**, *14*, 33-38; b) S. Manzetti, T. Lu, *J. Phys. Org. Chem.* **2013**, *26*, 473.

[13] A. Topacli, B. Kesimli, *Spectroscopy letters*, **2001**, *34*, 513.

[14] J. Jing, J. Yang, Z. Zhang, Y. Zhu, *Adv. Energy Mater.* **2021**, *11*, 2101392.

[15] F. Leng, H. Liu, M. Ding, Q.-P. Lin, H.-L. Jiang, *ACS Catal.* **2018**, *8*, 4583.

[16] E.S. Da Silva, N.M.M. Moura, M.G.P.M.S. Neves, A. Coutinho, M. Prieto, C.G. Silva, J.L. Faria, *Appl. Catal. B Environ.* **2018**, *221*, 56.

[17] T. He, S. Chen, B. Ni, Y. Gong, Z. Wu, L. Song, L. Gu, W. Hu, X. Wang, *Angew. Chem. Int. Edit.* **2018**, *57*, 3493.

[18] X. Fang, Q. Shang, Y. Wang, L. Jiao, T. Yao, Y. Li, Q. Zhang, Y. Luo, H.-L. Jiang, *Adv. Mater.* **2018**, *30*, 1705112.

[19] Q. Zuo, T. Liu, C. Chen, Y. Ji, X. Gong, Y. Mai, Y. Zhou, *Angew. Chem. Int. Ed.* **2019**, *58*, 10198.

[20] Y. Liu, L. Wang, H. Feng, X. Ren, J. Ji, F. Bai, H. Fan, *Nano Lett.* **2019**, *19*, 2614.

[21] J. Wang, Y. Zhong, L. Wang, N. Zhang, R. Cao, K. Bian, L. Alarid, R.E. Haddad, F. Bai, H. Fan, *Nano Lett.* **2016**, *16*, 6523.

[22] N. Zhang, L. Wang, H. Wang, R. Cao, J. Wang, F. Bai, H. Fan, *Nano Lett.* **2018**, *18*, 560.

[23] F. Le Formal, K. Sivula, M. Grätzel, *J. Phys. Chem. C* **2012**, *116*, 26707.

[24] a) P. Lefebvre, J. Allègre, B. Gil, H. Mathieu, N. Grandjean, M. Leroux, J. Massies, P. Bigenwald, *Phys. Rev. B* **1999**, *59*, 15363; b) G. Morello, F. Della Sala, L. Carbone, L. Manna, G. Maruccio, R. Cingolani, M. De Giorgi, *Phys. Rev. B* **2008**, *78*, 195313.

[25] X. Chen, J. Wang, Y. Chai, Z. Zhang, Y. Zhu, *Adv. Mater.* **2021**, *33*, 2007479.

[26] G. Wang, Q. Sun, Y. Liu, B. Huang, Y. Dai, X. Zhang, X. Qin, *Chem. Eur. J.* **2015**, *21*, 2364.

[27] E. Collini, C. Ferrante, R. Bozio, *J. Phys. Chem. C* **2007**, *111*, 18636.

[28] a) J. Rodriguez, C. Kirmaier, D. Holten, *J. Am. Chem. Soc.* 1**989**,*111*, 6500; b) G. E. O'Keefe, G. J. Denton, E. J. Harvey, R. T. Phillips, R. H. Friend, H. L. Anderson, *J. Chem. Phys.* **1996**, *104*, 805.
